# Colorimetric dual DNAzyme reaction triggered by loop-mediated isothermal amplification for the visual detection of Shiga toxin-producing *Escherichia coli* in food matrices

**DOI:** 10.1371/journal.pone.0320393

**Published:** 2025-04-23

**Authors:** Alaa H. Sewid, Joseph H. Ramos, Haley C. Dylewski, Gillian I. Castro, Doris H. D’Souza, Shigetoshi Eda

**Affiliations:** 1 School of Natural Resources, The University of Tennessee Institute of Agriculture, Knoxville, Tennessee, United States of America; 2 Department of Microbiology, Faculty of Veterinary Medicine, Zagazig University, Zagazig, Egypt; 3 Department of Microbiology, The University of Tennessee, Knoxville, Tennessee, United States of America; 4 Department of Chemical and Biomolecular Engineering, The University of Tennessee, Knoxville, Tennessee, United States of America; 5 Department of Molecular Physiology and Biophysics, Vanderbilt University, Nashville, Tennessee, United States of America; 6 Department of Food Science, The University of Tennessee Institute of Agriculture, Knoxville, Tennessee, United States of America; Yantai Institute of Technology, CHINA

## Abstract

Shiga toxin-producing *Escherichia coli* (STEC) is causing outbreaks worldwide and a rapid detection method is urgently needed. Loop-mediated isothermal amplification (LAMP) has attracted attention in the development of pathogen detection methods; however, current methods for the detection of LAMP amplicon suffer some drawbacks. In this study, we designed a new LAMP method by incorporating peroxidase-mimicking G-quadruplex DNAzyme for a simple colorimetric detection of the LAMP amplicon. As the new method produces LAMP amplicon containing two DNAzyme molecules per amplification unit, the method was termed colorimetric Dual DNAzyme LAMP (cDDLAMP). cDDLAMP was developed targeting 3 common STEC’s virulence genes (*stx1*, *stx2,* and *eae*) that are associated with serious human illnesses such hemorrhagic colitis and hemolytic-uremic syndrome. Immunomagnetic enrichment was used for specific, ultrasensitive, and fast detection of STEC in food samples (leafy vegetables and milk). The sensitivity of cDDLAMP ranged from 1–100 CFU/mL in pure culture to 10^0^–10^3^ CFU/mL in spiked milk, and 10^4^–10^9^ CFU/25g of lettuce. No cross-reaction with other generic *E. coli* strains and non-*E. coli* bacteria was observed. The color signal could be observed by the naked eye or analyzed by either UV–Vis spectra or smartphone platforms. Therefore, the cDDLAMP assay is a cost-effective method for detecting STEC strains without expensive machines or extraction methods.

## Introduction

Shiga toxin-producing *Escherichia coli* (STEC) is a significant cause of foodborne illnesses in the United States [1,2], with *E. coli* O157:H7 accounting for a substantial portion of cases and deaths [3–6]. Non-O157 STEC strains“ are also increasingly recognized for their clinical and economic impact [7–10].

Detecting STEC is challenging due to the low infectious dose [7,11]. Conventional culture-based methods, such as sorbitol-MacConkey agar, are time-consuming, labor-intensive, and non-specific for non-O157 STEC [12], even with chromogenic agars designed to differentiate non-O157 STEC from generic *E. coli*. Notably, 29.8% of STEC cases could not be cultured on MacConkey or CHROMagar STEC, highlighting limitations compared to PCR targeting the *stx* gene [13]. Quantitative polymerase chain reaction (qPCR) offers improved sensitivity and specificity with shorter testing time [14,15] but is not suitable for point-of-care (POC) use due to their complexity and cost.

Recently, loop-mediated isothermal amplification (LAMP), has emerged as a promising alternative for on-site diagnosis [16,17] offering high sensitivity, and tolerance to inhibitors in samples [17], which eliminates the need for DNA extraction kits [18,19].

Despite its potential, current LAMP assays for STEC detection [20–24] require expensive and/or sophisticated machines for real-time monitoring of turbidity or fluorescence signals [16], limiting their practicality for the use in resource-limited settings. Alternative end-point colorimetric LAMP designs were recently used for naked-eye detection of *E. coli* O157:H7 based on either pH-sensitive (phenol red) [25,26] or DNA-intercalating dyes (malachite green) [27]. However, these dyes are unsuitable for biological samples and require specific nucleic acid extraction kits because these dyes can trigger a color change without successful amplification, leading to false-positive results under certain conditions, such as pH fluctuations or the presence of other reactive components in the sample [28,29].

Short single-stranded DNA molecules with peroxidase-mimicking activity, collectively known named as G-quadruplex DNAzymes (simply called “DNAzyme” in this paper), have been reported to produce a stable and colored radical ion in the presence of H_2_O_2_, 2,2′-azino-bis (3-ethylbenzothiazoline-6-sulfonic acid) (ABTS), and intercalated hemin [30,31]. A recent study utilized DNAzyme in a colorimetric LAMP assay for *E. coli* O157:H7 detection using a molecular beacon for a “signal- off” approach [32]. However, a challenge with non-specific amplification arose due to the beacon’s recognition as a primer [33,34].

**Fig 1:**
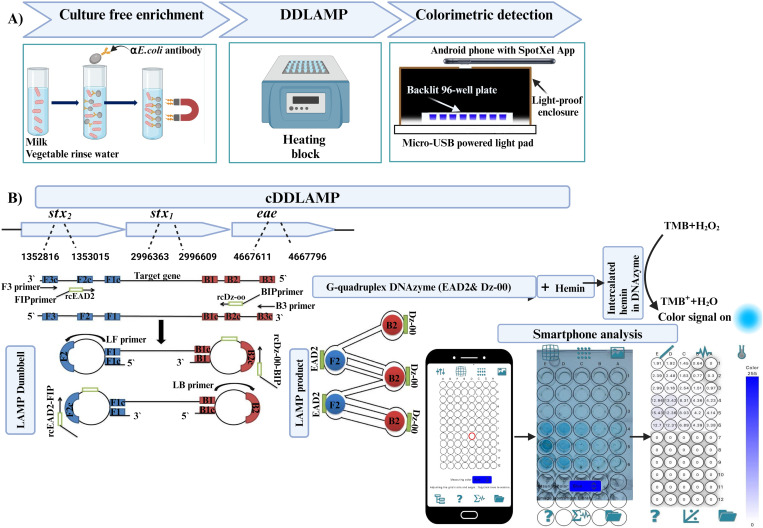
Illustrative depiction of incorporating dual DNAzyme mediated detection with LAMP amplification (cDDLAMP). (A) Overall operation of the cDDLAMP assay for the detection of STEC strains. Culture free enrichment: *E. coli* was enriched from milk samples and lettuce surface rinse water using magnetic beads functionalized with anti-*E. coli* (α-*E. coli*) antibodies. cDDLAMP: gDNA was extracted and amplified by LAMP in heat block to produce DNAzyme-containing LAMP amplicons. Colorimetric detection: The colorimetric signal produced by DNAzyme reaction was captured for analysis by using SpotXel Reader app on a smartphone. A micro-USB-powered light pad and a light-proof enclosure were used to eliminate the impact of ambient light. (B) cDDLAMP: Top panel shows a schematic illustration of target *stx1*, *stx2*, and *eae* genes of *E. coli* O157:H7. LAMP Dumbbell: The forward inner primer (FIP) and backward inner primer (BIP) were modified with reverse-complement sequence of DNAzyme (rcDNAzyme), rcEAD2 or rcDz-00. LAMP product: LAMP reaction generates amplicons containing two types of DNAzymes (EAD2, and Dz-00) in each amplification unit. Upon adding hemin, the DNAzymes produce a colorimetric signal in the presence of H_2_O_2_ and TMB. The color change is captured by smartphone camera, and analyzed with SpotXel, a smartphone-based microplate reader application.

Researchers have developed colorimetric LAMP assays for *Salmonella*, and *Listeria monocytogens* detection [35,36] by incorporation of Dz-00 DNAzyme sequence [37] in one of the inner primers, enabling color development in positive samples “signal- on” approach. However, the impact of incorporating other DNAzyme into LAMP assays remains unexplored. For example, EAD2 DNAzyme which has superior catalytic properties to all previously reported variants [38,39] has not yet been tested in DNAyzme-LAMP studies. In this study, we developed a novel colorimetric Dual DNAzyme reaction triggered by LAMP (cDDLAMP) assay for the detection of STEC strains targeting *stx1*, *stx2*, and *eae* genes by incorporating two different DNAzyme (EAD2, and Dz00) in both inner primers for LAMP reaction. The color signal analyzed using smartphone-based SpotXel reader and conventional microplate reader. Furthermore, our approach eliminates the need for culture-based enrichment, and complex DNA extraction by combining immunomagnetic concentration with a simple heat lysis of bacteria. A full depiction of our approach is presented in Fig 1A, which illustrates the enrichment of *E. coli* from milk and lettuce surface rinse water samples using streptavidin magnetic beads functionalized with anti-*E. coli* polyclonal antibodies (SMB-PAb). Following this enrichment step, genomic DNA (gDNA) of the enriched *E. coli* is extracted and amplified in heat block using the cDDLAMP method. This amplification generates dual DNAzyme-containing LAMP amplicons, which are then subjected to colorimetric detection. The new STEC detection method demonstrated a detection limit of 1–100 CFU/mL in pure culture, 10^0^–10^3^CFU/mL in spiked milk, and 10^4^–10^9^ CFU/25g of lettuce. Thus, the cDDLAMP method developed in this study has potential to be used for the monitoring of STEC outbreaks in resource-limited settings.

## Materials and methods

### Bacterial strains and genomic DNA extraction

STEC O157:H7 EDL932, a patient isolate from 1983 Michigan outbreak, was obtained from the American Type Culture Collection (ATCC 43894). Three clinical STEC strains (O111:H8, O26:H11, and O103:H11) were kindly provided by Dr. Pina Fratamico from USDA-APHIS (Beltsville, MD, USA), and were cultured at Department of Food Science, University of Tennessee (Knoxville, TN). Additionally, three other clinical STEC strains (O45:H2, O145:NT, and O121:H19) were obtained from STEC center at Michigan State University. Furthermore, three non-pathogenic reference *E. coli* strains (ATCC 43745, 25922, and K12), and four non-*E. coli* bacteria (*Salmonella cholerasuis* ATCC BAA 664, *Salmonella typhimurium* ATCC 14028, *Pseudomonas aeruginosa*, and *Staphylococcus aureus*) were provided by Department of Animal Science, University of Tennessee. Briefly, bacterial strains were cultured aerobically for 18 h at 37 °C in 5 mL of Tryptic Soy Broth (TSB, Becton Dickinson and Company, Franklin Lakes, NJ) in a shaking incubator at 225 rpm, and grown until the culture reaches confluency evaluated by the optical density (OD) at 600 nm. The OD was measured by using Ultrospec 10 (Biochrom, Cambourne, UK) and 1.0 OD at 600 nm was taken as 10^8^ CFU/mL. The bacterial pellet was harvested by centrifugation at 14,000 rpm for 2 min, and DNA was extracted using the Wizard Genomic DNA Purification Kit (Promega, Madison, WI) following manufacturer’s instructions. The DNA yield was determined spectrophotometrically by using NanoDrop 2200c spectrophotometer (Thermo Fisher Scientific, Waltham, MA). Alternatively, bacterial DNA was extracted by heat treatment. In brief, bacterial pellet was harvested by centrifugation at 14,000 rpm for 2 min, resuspended in Ultrapure DNase/RNase-free distilled water (Invitrogen), and heated at 99°C for 15 min in a heat block (Thermo Fisher Scientific, Waltham, MA). The bacterial suspension was centrifuged at 16,000 rpm for 5 min to sediment cell debris leaving extracted DNA in the supernatant. The extracted DNA was used as the genomic DNA (gDNA) template for cDDLAMP assays.

### LAMP primer modification, and optimization

Two Shiga-toxin-producing virulence genes (*stx1*and *stx2*) and intimin gene (*eae* gene) were selected as detection targets in this study. LAMP primers were adapted from previously published papers [23,40]. Another two primer sets targeting *stx2* gene with different distance between the F3 and B3 (229 bp, and 214 bp) were adapted from previously published papers [23,41]. As shown in Fig 1B, LAMP primers target *stx2*, *stx1*, and *eae* gene sequences of *E. coli* O157:H7 EDL933 (accession: NC_002655, region: 1352816–1353015, 2996363–2996609, and 4667611–4667796, respectively). Oligonucleotide primers were obtained from Integrated DNA Technologies (IDT, Coralville, IA), and their sequences were shown in S1 Table. The forward inner primer (FIP) and backward inner primer (BIP) were modified with the target recognition site at 3′ end (F2 and B2), and target complementary site at 5′ end (F1c and B1c) were linked with reverse-complement sequence of DNAzyme (rcDNAzyme) (Fig 1B, and S1 Fig). Two different DNAzymes (EAD2, and Dz-00) [37,39] were used to modify FIP and BIP, respectively (Fig 1B, and S1 Fig). The normal primers without rcDNAzyme sequence served as control primer set (control primers) (S1 Fig).

The primer stocks (10×) used in this study were composed of 2 μM outer primers (F3 and B3), varying concentrations of loop primers (LB and LF, from 4 to 6 μM), and varying concentrations of inner primers (FIP, and BIP, from 8 to 16 μM). The optimized reaction mixture (10 μL) contained 5 μL of WarmStart LAMP 2× Master Mix (New England Biolabs), 1 μL of 10× primer stocks, 1 μL of gDNA template (at 1 ng/reaction=1.67 × 10^5^ copies/reaction), and nuclease-free water (Thermo Fisher Scientific, Waltham, MA). To further improve assay performance, we modified the LAMP reaction by adding 0.8 M betaine (Thermo Fisher Scientific, Waltham, MA) and/or 320 U/mL *Bst* 3.0 DNA polymerase (New England Biolabs). The mixture was incubated at 65 °C in a heating block (Thermo Fisher Scientific, Waltham, MA) for 30–40 min. At the end of the amplification phase, the LAMP reaction was inactivated by heating the reaction mixture at 95 °C for 2 min. Reaction mixture without DNA extract (to account for amplicon carryover contamination) was included as a negative control (termed no templated control, NTC). Both the DNAzyme-LAMP products and normal LAMP products were verified using gel electrophoresis on a 1% agarose gel containing ethidium bromide. The electrophoresis was carried out at 80 V for 30 min, and the LAMP products were visualized under a UV transilluminator (UVP UVsolo Touch).

### cDDLAMP procedure

The cDDLAMP procedure consisted of a LAMP reaction followed by formation of G-quadruplex DNAzymes within the LAMP products and reaction of their reaction with 3,3,5,5-tetramethylbenzidine (TMB) as shown in Fig 1B. A10 μL LAMP reaction mixture was heated at 95 ºC for 5 min, then cooled at room temperature, followed by the addition of 1.1 μL of hemin (200 μM in dimethyl sulfoxide [DMSO] MP Biomedicals). Temperature (25°C-95°C) and time (5 min-20 min) required for G-quadruplex DNAzyme folding in the LAMP amplicon were optimized. After the hemin incubation with LAMP amplicon, 50 μL of a TMB solution (TMB Substrate Kit, Thermo Fisher Scientific, Waltham, MA) was added to the LAMP reaction mixture and incubated at room temperature for 0–12 min. An Android smartphone was used to capture the image of reaction mixture in wells of a 96 well-plate, and the color intensity of each well was analyzed with SpotXel [42] or Model 680 plate reader at 655 nm (BioRad, Hercules, CA).

### Evaluation of specificity and sensitivity of cDDLAMP

The specificity and sensitivity of LAMP targeting *stx1*, *stx2* and *eae* genes were previously evaluated [23,40]. For verification of the specificity of our cDDLAMP system, *E.coli* O157:H7 EDL932 (ATCC 43894) was used as a positive control strain, while three non-pathogenic *E.coli* strains (ATCC 43745, 25922, and K12) and four non-*E. coli* bacteria (*Salmonella cholerasuis* ATCC BAA 664, *Salmonella typhimurium* ATCC 14028, *Pseudomonas aeruginosa*, and *Staphylococcus aureus*) were used as negative controls. Reaction mixture without target bacteria was used as another negative control and termed no-template control (NTC). Six clinical STEC strains O111:H8, O26:H11, O103:H11, O45:H2, O145:NT, and O121:H19 were also used for the evaluation of cDDLAMP system. cDDLAMP products were analyzed by agarose gel electrophoresis and by DNAzyme reaction as described above. To evaluate the sensitivity of cDDLAMP method, serial 10-fold dilutions of *E. coli* O157:H7 EDL932 (ATCC 43894) (1.67× 10^2−0^ CFU/mL) were used to make bacterial lysates for crude DNA extraction as described above.

### PCR amplification and sequencing

As a reference test, PCR amplification of *stx1*, *stx2*, and *eae* genes of STEC strains (O157:H7, O111:H8, O26:H11, O103:H11, O45:H2, O145:NT, and O121:H19) were carried out in C1000 thermal cycler (BioRad, CA). The PCR reaction mixture (25 μL) contained 1× PowerUp SYBER MasterMix (Applied Biosystems. Waltham, MA), F3 and B3 LAMP primers (0.4 μM each), and genomic DNA (10 ng). The PCR reaction was conducted with 1 min initial denaturation at 95°C followed by 35 cycles of denature (95°C, 15 sec), annealing (50°C, 30 sec) and extension (60°C, 1 min). The PCR products (247, 258, and 203 bp, respectively) were then confirmed by 1.5% agarose gel electrophoresis. PCR products were purified using the QIAquick PCR Purification Kit (Qiagen, Hilden, Germany) and sequenced at the University of Tennessee, Knoxville, Genomics Core Facility. Gene sequences were aligned and compared using Geneious software (Biomatters, Auckland, New Zealand).

### Detection of *E. coli* O157:H7 in lettuce surface rinse water

Romaine lettuce was purchased from a retail store, immediately transported to the laboratory with proper storage conditions, and used for artificial inoculation by *E. coli* O157:H7 as previously described [43]. Before the inoculation, lettuce sample was cut into small pieces (approximately 4 cm^2^/piece), rinsed with deionized water, and irradiated with ultraviolet in a BSL-2 biosafety cabinet for 10 min on each side of the lettuce to eliminate the background microorganisms.

Approximately 1 g of the lettuce sample was placed into a Whirl-Pak homogenizer blender filter bag (Nasco, Fort Atkinson, WI), and 10 mL of the inoculum suspension was added in each bag. The inoculum contained approximately 5.2 ×10^5^, 5.2 × 10^3^, or 5.2 × 10^0^ CFU/g as previously described [44,45]. Three lettuce samples tested to confirm absence of *E. coli* O157:H7 using Sorbitol MacConkey agar (SMAC) culturing methods and used as uninoculated controls [46]. The inoculated lettuce was placed into a sterile TSB (5 mL), and the lettuce surface rinse water was prepared by vigorous shaking at 115 rpm for 10 min. *E. coli* in the rinse water was enriched by using anti-*E. coli* antibody-coated magnetic beads. In brief, 50 μL of streptavidin magnetic beads (SMB, New England Biolabs, Ipswich, MA) were modified with 5 μL of biotinylated anti-*E. coli* polyclonal antibody (5 mg/mL with 0.1% sodium azide, Invitrogen) for 10 min at 37°C. The antibody-coated SMB (SMBs-PAb) was added to the lettuce surface rinse water and kept at 37 °C for another 30 min without shaking. The SMBs-PAb-*E. coli* complexes were collected for 1 min on a magnetic bead separation rack (Thermo Fisher Scientific, Waltham, MA). Then, the SMB pellet was washed twice with 500 μL of PBST (1x PBS with 0.05% Tween 20, pH 7.2) and resuspended in 100 μL of PBST. For downstream cDDLAMP assay, genomic DNA (gDNA) was extracted from each dilution via heat lysis as described above.

For detection of *E. coli* O157:H7 on large volume of lettuce, aliquots of 1.5 ml of the inoculum suspension were applied across abroad range of concentrations (10^3-9^ CFU) on 25 g of lettuce, approximately equivalent to 10^¹^–10^7^ CFU/g). Within this range, 10^³^–10^4^ CFU/25 g (approximately 10^¹^–10^2^ CFU/g) is categorized as a “low-level inoculation”. The inoculum was equally divided in small volumes and inoculated at 5 to 10 locations on the lettuce sample to facilitate drying as described previously [24,47,48]. Three lettuce samples tested to confirm absence of *E. coli* O157:H7 using SMAC culturing methods and used as uninoculated controls [46]. The inoculated lettuce was transferred to filter Whirl Pak bags (1.6 mL) as described previously [49–51], and lettuce surface rinse water was prepared by addition of 225 ml of buffered peptone water (BD Diagnostic Systems, Sparks, MD) followed by shaking at room temperature at 185 rpm. Aliquots (1 mL) of each sample were centrifuged at 16,000 rpm for 3 min, and pellets were suspended in 100 µL of Ultrapure DNase/RNase-free Distilled Water (Invitrogen). Genomic DNA was extracted from the bacterial pellet by heat lysis as described above. Another aliquot (5 mL) of each sample was used for enrichment of *E. coli* with SMBs-PAb as described above for genomic DNA extraction via heat lysis.

### Detection of *E. coli* O157:H7 in milk samples

Aliquots (5 mL each) of commercial pasteurized bovine whole milk were artificially spiked with bacteria at different cell densities: 5.2 × 10^5^, 5.2 × 10^3^, and 5.2 × 10^0^ CFU/mL [52]. The spiked milk samples were diluted with 5 mL of PBST at 1:1 ratio. *E. coli* in the spiked sample was enriched by using SMBs-PAb and used for gDNA extraction.

### Statistical analysis

A two-way ANOVAs was performed to determine if there were a significant difference in the signal to noise ratio (S/N) of the cDDLAMP of all target genes (*stx1*, *stx2*, and *eae* genes), and a one-way ANOVA was performed to make a single comparison of 3 different primer sets targeting *stx2* gene. All post hoc tests used Tukey’s honestly significant difference (HSD) method. Data represents mean values from three independent experiments, and with error bars indicating standard deviations. A p-value <0.05 was considered significant. The cut-off value represented the upper bound of 95% confidence interval for negative samples (mean + 2SD) [53]. The correlation analyses and visualization were done using JMP Pro, Version 17. OS (64-bit) to provide an estimate for the association among the SpotXel and microplate reader. All analyses were conducted using JMP Pro, Version 17. OS (64-bit). All graphs were prepared using the GraphPad Prism software (Version 8, GraphPad Software Inc.).

## Result

### Optimization of reaction conditions, and validation of cDDLAMP system

The LAMP reaction conditions were optimized by testing different concentrations of primers, different amplification times, and by adding 0.8 M betaine and 320 U/mL *Bst* polymerase 3.0 to the reaction mixture. The reaction temperature of DNAzyme was also optimized.

**Fig 2:**
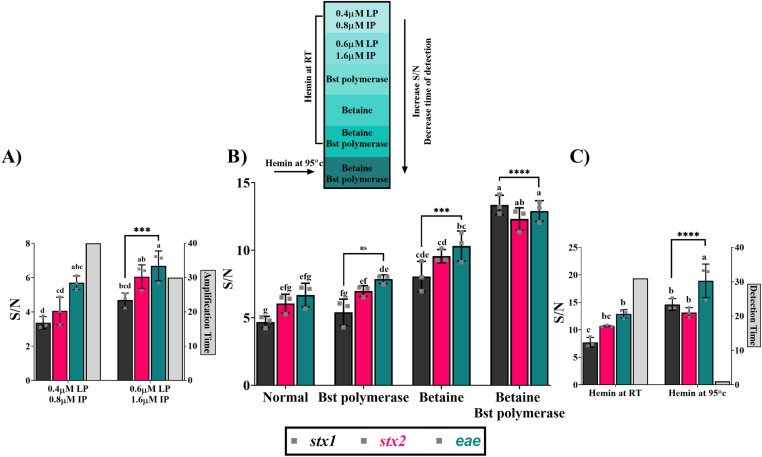
Optimization of cDDLAMP reaction conditions for the detection of *stx1, stx2*, and *eae* genes of *E. coli* O157:H7. Heat map showing the impact of primer concentration, master mix, and temperature modifications on the signal to noise ratio (S/N), and detection time. The modifications, applied progressively from top to bottom, showed improvements in S/N and reduced detection time. All reactions were evaluated by colorimetric measurement, and data from three independent experiments are shown with error bars representing standard error of the means. (A) Increased primer concentrations. Shown are identical reactions differing only in the concentration of inner primers (FIP/BIP, 0.8-1.6 µM), and loop primers (LF/LB, 0.4-0.6 µM). The value on the right x-axis signifies the amplification time (30 and 40 min). a–d; Means carrying different superscripts are significantly different at p < 0.05 (***P=0.0004). (B) Supplementation of NEB WarmStart reaction mix with betaine (0.8 M) and/or *Bst3.0* polymerase 3 (320 U/ml) compared with normal NEB WarmStart” reaction mix. a–g; Means carrying different superscripts are significantly different at p < 0.05 (***P < 0.0008, ****P < 0.0001, and ns p > 0.05). (C) The effect of reaction temperature (95°C or room temperature [RT]) upon hemin interaction with active DNAzymes (EAD2 and Dz-00). The value on the right x-axis signifies the detection time (1 and 30 min). a–c; Means carrying different superscripts are significantly different at p < 0.05 (****p < 0.0001).

While optimizing the concentration of primers to improve assay performance, we found that increasing concentrations of loop primers (from 0.4 to 0.6 µM) and inner primers (from 0.8 to 1.6 µM) improved the amplification time from 40 min to 30 min while increasing the S/N of all target genes (p=0.0004) (Fig 2A, S2A Fig). Next, we modified the LAMP mixture by supplementing the Warmstart master mix with betaine (0.8 M) and/or *Bst* polymerase 3.0 (320 U/ml) and assessed the improvements in amplification efficiency. A progressive increase of the S/N was observed with all target genes*, stx1*, *stx2*, and *eae* (p<0.0001), with the addition of *Bst* polymerase and betaine. The addition of betaine alone to the reaction mixture led to a noticeable increase in the S/N of all target genes, *stx1*, *stx2*, and *eae* (p=0.0008, 0.0005, and 0.0003, respectively). On the other hand, *Bst* polymerase addition alone did not significantly change the S/N of all target genes, *stx1*, *stx2*, and *eae* (p=0.989, 0.934, and 0.765 respectively) (Fig 2B, S2B Fig).

Increasing the temperature for DNAzyme-hemin complex formation from 25ºC to 95ºC improved the S/N of all target genes, *stx1*, *stx2*, and *eae* (p<0.0001), while shortening the detection time from 30 min to 1 min (Fig 2C, S2C Fig).

Different primer sets targeting *stx2* gene were tested for the cDDLAMP assay. Among these, the primer set with a 258 bp distance between F3 and B3 demonstrated a significantly higher S/N (p < 0.0001) compared to the other two primer sets (p > 0.05) (S3 Fig). Consequently, the primer set with the 258 bp distance between F3 and B3 was selected for use in subsequent experiments.

### Feasibility of cDDLAMP

**Fig 3:**
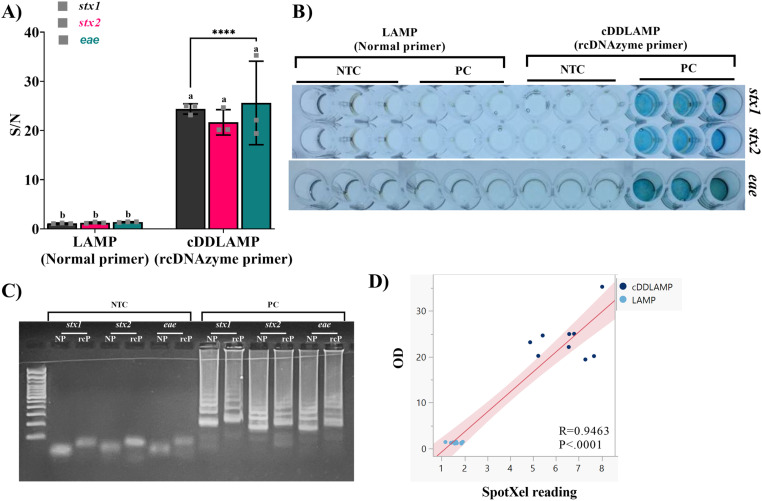
Detection of *stx1*, *stx2*, and *eae* genes with the optimized cDDLAMP. (A) Signal to noise ratio (S/N) of the cDDLAMP targeting *stx1*, *stx2*, and *eae* genes compared with LAMP normal primers. Means from three independent experiments are shown with error bars representing standard error of the means. a–b; Asterisks above means denote significant differences and means carrying different superscripts are significantly different at p < 0.05 (****P < 0.0001). (B) A representative image of color development in a 96-well plate. (C) Gel electrophoresis image of typical cDDLAMP amplicons using rcDNAzyme primers (rcP) compared with LAMP amplicons with normal primers (NP). Scatter plot showing the correlation coefficient and probability (R = 0.9463, p <.0001) between data obtained by SpotXel (x-axis) and microplate reader (y-axis, OD: optical density at 655 nm). (D) The shaded zone represents the 95% confidence interval of the trendline.

Shiga toxin genes (*stx1*, *stx2*, and *eae*) of *E. coli* O157:H7 were detected with the optimized cDDLAMP to verify the feasibility of the assay. The S/N of the cDDLAMP reaction with rcDNAzyme primers exhibited a significantly higher colorimetric signal for all target genes, *stx1*,*stx2*, and *eae* (p<0.0001), compared to the LAMP reaction containing normal primers without rcDNAzymes (Fig 3A). As seen in the image of the 96-well plate (Fig 3B), the amplification of *E. coli* O157:H7 genes by cDDLAMP reactions resulted in a clearly visible dark blue color change in the presence of target genes, indicating the feasibility of naked-eye detection. Furthermore, an increase in cDDLAMP amplicon size due to the incorporation of two different DNAzyme sequences (EAD2 & Dz-00) was clearly observed when visualized using agarose gel electrophoresis (Fig 3C). As shown in Fig 3D, the SpotXel reader and microplate reader readouts positively correlated (R = 0.9463) with a significant correlation probability (P<.0001), and an excellent linear relationship OD=−5.160147+4.3614846xSpotXel.

### Evaluation of cDDLAMP`s specificity, and sensitivity for STEC detection

**Fig 4:**
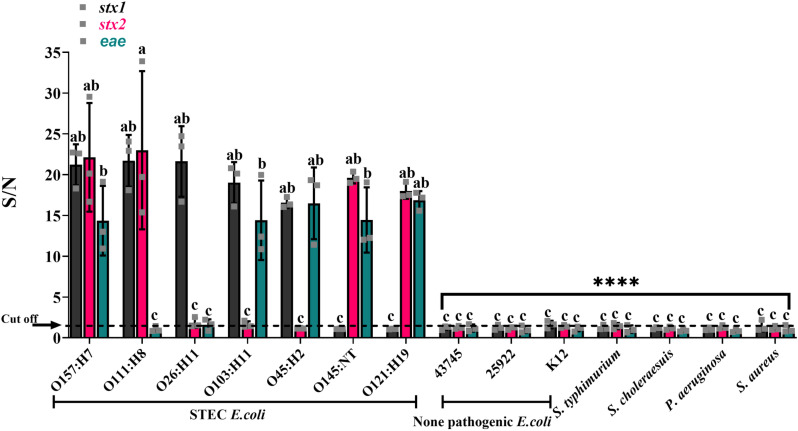
Specificity evaluation of cDDLAMP of all target genes (*stx1, stx2*, and *eae* genes). The following bacterial species were tested: O157:H7 EDL932 (ATCC 43894), six clinical STEC strains (O111:H8, O26:H11, O103:H11, O45:H2, O145:NT, and O121:H19), three non-pathogenic *E. coli* strains(ATCC 43745, 25922,and K12), and four non-*E. coli* bacteria (*Salmonella cholerasuis* ATCC BAA 664, *Salmonella typhimurium* ATCC 14028, *Pseudomonas aeruginosa*, and *Staphylococcus aureus*). Means from three independent experiments are shown with error bars representing standard error of the means. a–c; Asterisks above means denote significant differences and means carrying different superscripts are significantly different at p < 0.05 (****P < 0.0001). The cut-off value of *stx1* indicated as dotted line = 1.45. The *stx2* and *eae* genes were 1.37and 1.18, respectively.

For the specificity evaluation of cDDLAMP system, gDNA of O157:H7 EDL932 (ATCC 43894), six clinical STEC strains (O111:H8, O26:H11, O103:H11, O45:H2, O145:NT, and O121:H19), three non-pathogenic *E. coli* strains (ATCC 43745, 25922,and K12), and four non-*E. coli* bacteria (*Salmonella cholerasuis* ATCC BAA 664, *Salmonella typhimurium* ATCC 14028, *Pseudomonas aeruginosa*, and *Staphylococcus aureus*) were tested. No cross reaction with the non-pathogenic *E. coli* strains nor non-*E. coli* bacteria were observed with the S/N less than the cut-off value, whereas all STEC strains displayed a significant higher S/N for their harboring toxin gene(s) than the cut-off value (p<0.0001) (Fig 4). A representative image of the color signal are indicated (S4, and S5A Figs). These data were confirmed by agar gel electrophoresis (S4, and S5B Figs).

**Fig 5:**
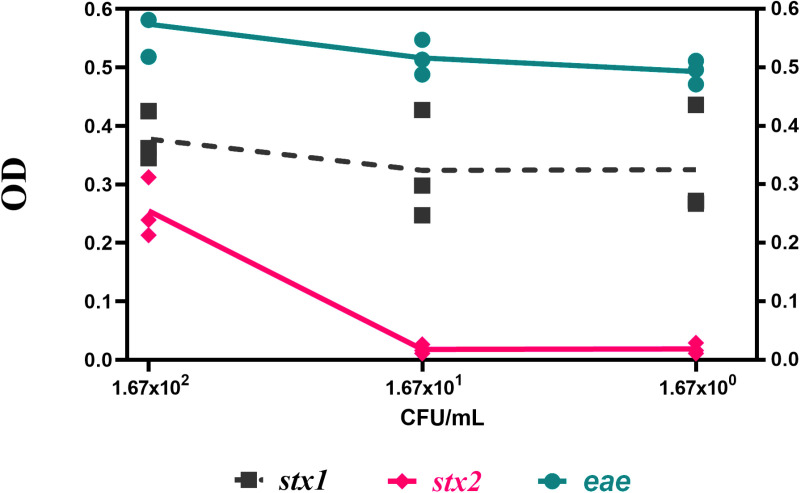
The sensitivity of cDDLAMP for *stx1, stx2*, and *eae* genes. The serial 10-fold dilutions of O157:H7 EDL932 (ATCC 43894) (1.67 × 10^0-2^ CFU/mL) were used in this experiment. The values represent the average from three independent experiments, and the individual data points are shown. OD: optical density at 655 nm.

To verify the sensitivity of the cDDLAMP method, DNA extracted from serial 10-fold dilutions of O157:H7 EDL932 (ATCC 43894) (1.67×10^2−0^ CFU/ml) were used as templates. The *stx1* and *eae* DDLAMP assays consistently detected down to 1.67 × 10^0^ CFU/mL. However, *stx2* cDDLAMP was 100-fold less sensitive. Tentative LODs of cDDLAMP for *stx1*, *stx2*, and *eae* genes were found to be 1.67 × 10^0^ CFU/mL, 1.67× 10^2^ CFU/mL, and 1.67× 10^0^ CFU/mL, respectively (Fig 5).

### PCR detection and sequence analysis

The toxin genes in O157:H7, O111:H8, O26:H11, O103:H11, O45:H2, O145:NT, and O121:H19 were confirmed to be *stx1*^+^/*stx2*^+^/*eae*^+^, *stx1*^+^/*stx2*^+^/*eae*^-^, *stx1*^+^/*stx2*^-^/*eae*^-^, *stx1*^+^/*stx2*^-^/*eae*^+^, *stx1*^+^/*stx2*^-^/*eae*^+^, *stx1*^-^/*stx2*^+^/*eae*^+^, and *stx1*^-^/*stx2*^+^/*eae*^+^ respectively (S5C, and S6 Figs).

**Fig 6:**
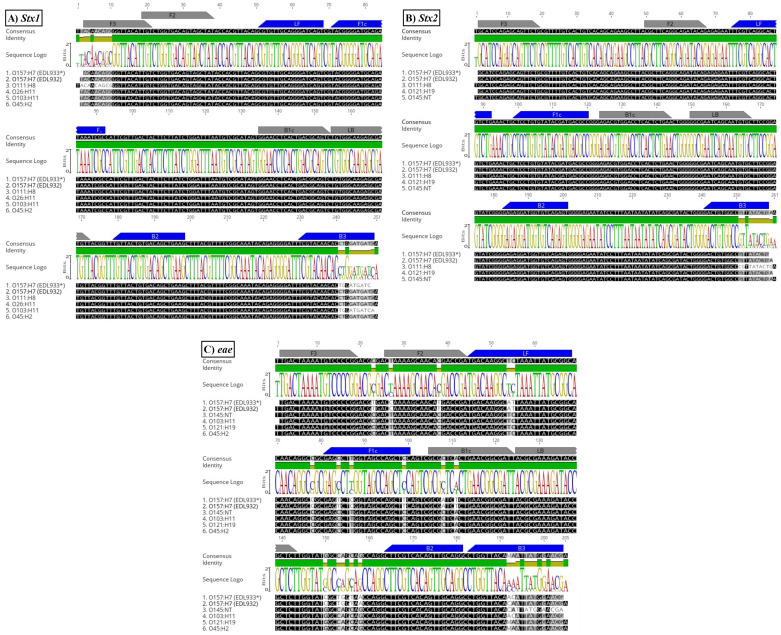
Primer binding regions for *Stx1*, *Stx2*, and *eae* genes. ClustalW alignment of the *stx1* (A), *stx2* (B), and *eae* (C) genes from STEC strains is presented. Target sequences for *stx1*, *stx2*, and *eae* from *E. coli* O157:H7 (strain EDL933*) were retrieved from the GenBank database (accession number CP008957.1). Annotation labels indicate the binding regions for the primers, including outer primers (F3, B3), loop primers (LF, LB), forward inner primer (FIP; F1c, F2), and backward inner primer (BIP; B1c, B2). Shading indicates sequence similarity as follows: black (100%), dark gray (80–100%), light gray (60–80%), and white (<60%). Sequence logos, generated using Geneious 2019.2.1 software, display the consensus across the sequences.

DNA sequencing validated the specificity of cDDLAMP by confirming the identity of the amplified fragments and ensuring they correspond to the target toxin genes (*stx1*, *stx2*, or *eae*) without evidence of nonspecific amplification. Sequence alignments displayed homologies indicated by shading: black (100%), dark gray (80–100%), light gray (60–80%), and white (<60%), in comparison to the reference sequence of *E. coli* O157:H7 (strain EDL933*; accession number CP008957.1), as illustrated in Fig 6.

### Rapid detection of *E. coli* O157:H7 in spiked lettuce by cDDLAMP

**Fig 7:**
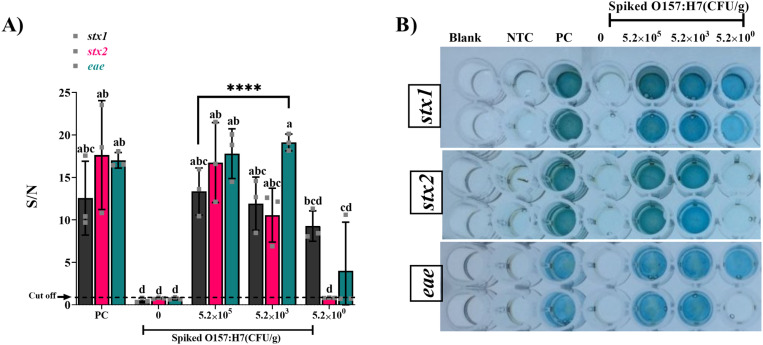
cDDLAMP detection of *E. coli* O157:H7 inoculated on lettuce (5.2 ×10^0-5^ CFU/g). gDNA was extracted from recovered *E. coli*, amplified with cDDLAMP, and analyzed colorimetrically (A). Results represent means from three independent experiments, with error bars representing standard error. Significant differences (p < 0.05) are denoted by asterisks (****P < 0.0001) and different superscripts (a–d). The cut off value of *stx1* indicated as dotted line = 1.18. The *stx2* and *eae* genes were 0.96 and 1.06, respectively. A photograph of representative plates is shown in (B). NTC: no template control; PC: positive control.

*E. coli* (5.2 ×10^0-5^ CFU/g) in lettuce surface rinse water was recovered by SMB-PAb enrichment. gDNA was extracted from *E. coli* and amplified using cDDLAMP. The resulting amplicons were analyzed through colorimetric detection. All target genes (*stx1*, *stx2*, and *eae*) were detected by cDDLAMP down to 5.2×10^3^ CFU/g lettuce (p<0.0001) (Fig 7). For the uninoculated controls, all target genes were tested negative by cDDLAMP, confirming the absence of false positive reactions. Notably, the *stx1* cDDLAMP consistently detected *E. coli* O157:H7 at an even lower concentration (5.2 ×10^0^ CFU/g) with all three replicates above the cut-off value, 1.18. On the other hand, *stx2* cDDLAMP was less sensitive, with all three replicates of the lower concentration (5.2×10^0^ CFU/g) under the cut off value of 0.957. For *eae* gene, cDDLAMP could detect one out of three replicates (above the cut off value of 1.06) at 5.2×10^0^ CFU/g.

**Fig 8:**
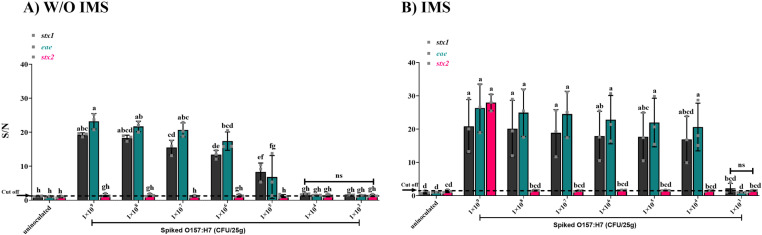
cDDLAMP detection of E. coli O157:H7 artificially inoculated on lettuce (10^3-9^ CFU/25 g). gDNA of the *recovered E. coli* without SMB-PAb enrichment (A) or with SMB-PAb enrichment (B) was amplified with cDDLAMP. In (A), the cut off value of *stx1*, and *eae* genes indicated as dotted line = 1.44. In (B), the cut off value of *stx1/2* and *eae* genes (dotted line) were 2.08 and 1.12, respectively. Means from three independent experiments are shown with error bars representing standard error. Significant differences (p < 0.05) denoted by different superscripts (a–h). n.s.: Not significant (ns p > 0.05).

Furthermore, the sensitivity of cDDLAMP was evaluated by using a large volume of lettuce that had been spiked with low- level inoculation down to10^3^ CFU/25g (Fig 8A and S7A Fig). In this experiment, enrichment with SMBs-PAb was not included. For the uninoculated controls, all target genes (*stx1*, *stx2*, and *eae*) were tested negative by cDDLAMP. The *stx1* cDDLAMP consistently detected *E. coli* O157:H7 down to 10^5^ CFU/25g lettuce with all three replicates above the cut off value of 1.44. In comparison, *eae* cDDLAMP could be detected in two out of three replicates (above the cut off value of 1.44) down to approximately 10^5^ CFU/25g. Notably, the cDDLAMP assay of all target genes failed to detect the *E. coli* O157:H7 at 10^3-4^ CFU/25g lettuce (p>0.05). It is also worth mentioning that *stx2* cDDLAMP failed to detect *E. coli* O157:H7 in large volume of spiked lettuce.

To improve the sensitivity, immunomagnetic separation (IMS) using SMBs-PAb was conducted prior to cDDLAMP reaction. As shown in Fig 8B, 10^4^ CFU/25g of O157:H7 could be detected by *stx1* and *eae* cDDLAMP with all three replicates above the cut off value of 2.08, and 1.12, respectively. *Stx2* cDDLAMP could detect 10^9^ CFU/25 g of O157:H7 (Fig 8B) which could not be detected without IMS (Fig 8A). Photographic images of this assay were shown in S7B Fig.

### cDDLAMP detection of *E. coli* O157:H7 in spiked raw milk sample

**Fig 9:**
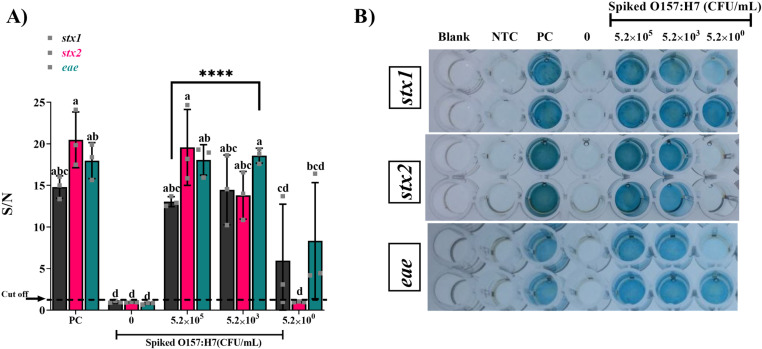
cDDLAMP E. coli O157:H7 artificially inoculated in milk. gDNA of *E. coli* was extracted and amplified with cDDLAMP. The cDDLAMP amplicons were reacted with TMB, and the signal to noise ratio (S/N) was calculated (A). A representative photograph of well plate is shown in (B). Means from three independent experiments are shown with error bars representing standard error. Significant differences (p < 0.05) are denoted by asterisks (****P < 0.0001) and different superscripts (a–d). The cut off value of *stx1* indicated as dotted line = 1.29. The *stx2* and *eae* genes were 1.05 and 1.10, respectively.

cDDLAMP was evaluated for the detection of *E. coli* O157:H7 in raw milk by spiking the samples with 5.2×10^0-5^ CFU/mL*. E. coli* was enriched from milk samples by using SMB-PAb (Fig 9). For the uninoculated controls, all target genes (*stx1*, *stx2*, and *eae*) were tested negative by cDDLAMP. Milk samples containing O157:H7 at the cell density of 5.2×10^3^ or 5.2×10^5^ CFU/mL consistently produced a significant signal for all target genes (*stx1*, *stx2*, and *eae*) (p < 0.0001) with all three replicates above the cut off value of 1.29, 1.05, and 1.10, respectively. The LOD of *stx1* and *eae* cDDLAMP could reach 5.2×10^0^ CFU/mL, but there was a high variation of the signals.

### Use of smartphone app, SpotXel, in cDDLAMP assay

Use of a smartphone app for analysis of color change will allow users to conduct cDDLAMP assay without the need of a relatively bulky and expensive microplate reader. In the following experiments, a smartphone app, SpotXel, was evaluated as an alternative method for the microplate reader which was used in the experiments above. Photographic images of experiments shown in Figs 7–9 were analyzed by using SpotXel and plotted against OD values obtained by a microplate reader.

**Fig 10:**
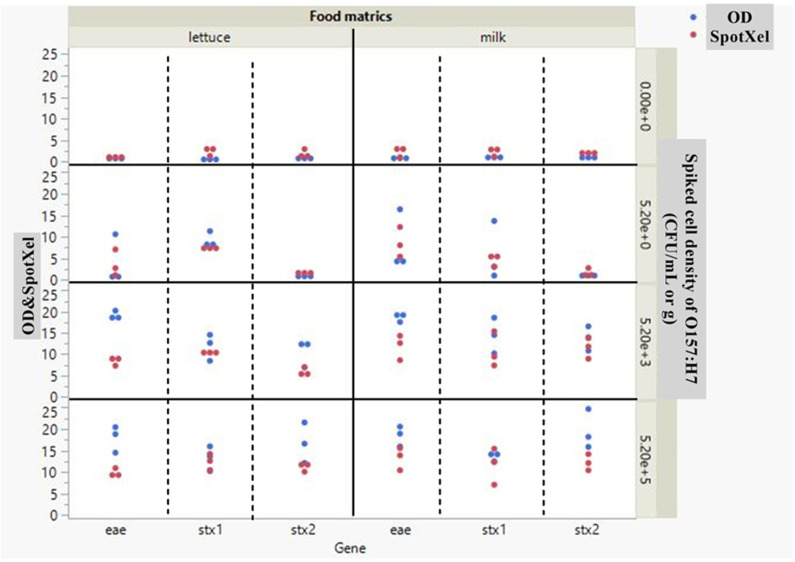
Scatter plot comparing the signal to noise ratio (S/N) of SpotXel reader and microplate reader (OD 655nm) (y-axis) with cDDLAMP target genes *eae*, *stx1*, and *stx2* (x-axis). The food matrices (milk and lettuce) artificially inoculated with *E. coli* O157:H7 at 5.2 x 10^5,3,0^ CFU/mL or g. Red and blue data points represent SpotXel reader and microplate reader (OD 655 nm), respectively. Each dot indicates a single data point. The data for the plots are appended in S2A Table.

**Fig 11:**
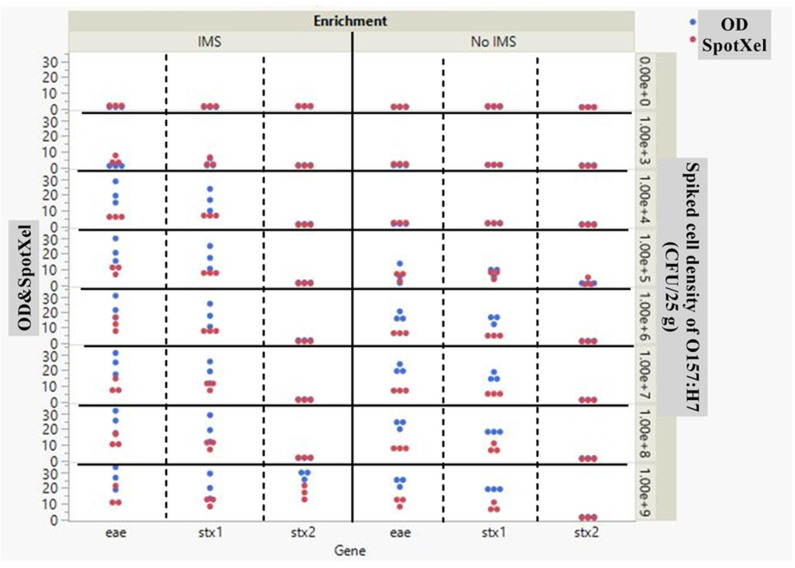
Scatter plot comparing the effects of IMS on the signal to noise ratio (S/N) of SpotXel reader and microplate reader (OD655nm) (y-axis) with cDDLAMP target genes *eae, stx1*, and *stx2* (x-axis). Lettuce was artificially inoculated with *E. coli* O157:H710^9-3^CFU/25g. *E. coli* were recovered from the lettuce with IMS (IMS) or without IMS (No IMS). Recovered *E. coli* was subjected to cDDLAMP. Red and blue data points represent SpotXel reader and microplate reader (OD 655 nm), respectively. Each dot indicates a single data point. The data for the plots are appended in S2B Table.

Based on the data obtained by cDDLAMP assay for the detection of *stx1*, *stx2,* and *eae* genes of O157:H7 in food matrices, it was found that the optical density data obtained by SpotXel and microplate reader had a positive correlation with a correlation coefficient (R) of 0.8675, and the linear equation derived from this relationship was OD=−0.397977+1.3976621xSpotXel (Fig 10, S2A Table). Similarly, based on the data obtained by cDDLAMP testing of spiked lettuce samples with and without IMS, the correlation coefficient (R) and the linear equation derived from this relationship were 0.8605 and OD=−0.086102+2.1565284xSpotxel respectively (Fig 11, S2B Table).

## Discussion

Point-of-Care (POC) methods with high specificity and sensitivity are crucial for monitoring food sources for STEC [54]. Among food commodities, raw milk is associated with isolated cases and extensive global foodborne outbreaks linked to STEC [55–57]. STEC causes approximately 30% of produce-related disease outbreaks, ranking second only to norovirus [58–62]. This public health concern is largely due to the consumption of raw produce with insufficient cleaning [63,64]. Detecting STEC contamination in lettuce is critical because eight foodborne outbreaks have been reported in the United States [65], and 26 cases were confirmed in the United States in 2010 [66].

Several studies highlight the challenges in STEC detection in milk and leafy vegetables due to the presence of inhibitory compounds (such as chlorophylls and polysaccharides) [67–69], very low levels of STEC contamination, and interfering background microbiota [70,71]. The challenge of isolating the target pathogen increases due to the existence of microbiota that interferes with culture-based STEC detection [72,73]. This issue is worsened especially when the enrichment period is extended to 12 or 24-hours for milk and lettuce, respectively, as recommended in the FDA BAM protocol [74,75]. Thus, the implementation of immunomagnetic separation (IMS) would be an effective tool for enhancing the STEC recovery from complex food matrices that contain high background microbiota [76–79].

Simultaneous detection of common virulence genes (*stx1*, *stx2*, and *eae*) is important due to the growing clinical importance of non-O157 STEC strains worldwide [4] beside the most relevant STEC serotypes, *E. coli* O157:H7 [80]. STEC genomes contain at least one Shiga-toxin (*Stx*) encoding genes (*stx1* or *stx2*) [81]. DNAzyme-modified inner primers can be tailored to detect various target genes without compromising assay specificity [82]. In this study, we validated the novel cDDLAMP approach by employing a previously established LAMP primer set, which has been verified for its specificity and sensitivity in detecting *stx1*, *stx2*, and *eae* genes [23,40]. Our results showed that the *stx1* and/or *stx2* genes could be successfully detected by cDDLAMP assay in all tested STEC strains without false positive reaction with non-STEC *E. coli* strains or non-*E. coli* bacteria. The *eae* gene has been reported to cause human illness [83]. The presence of *eae* gene in *E. coli* O157:H7 and O103:H11 was reported in previous studies using LAMP assays [23,40] and confirmed in this study using cDDLAMP method. However, our assay did not detect the *eae* gene in O26:H11 and O111:H8 strains, despite these serotypes typically being associated with specific *eae* subtypes (e.g., β1 and θ) [84–86]. This may indicate that not all strains within these serotypes consistently harbor the *eae* gene, as *eae*-negative strains of the O111 serotype have been reported in previous studies [23,87]. Alternatively, the primers used in this study, designed based on previous study to target the *eae-*γ*1* subtype associated with the O157:H7 serotype [23], may have limited the detection of other *eae* subtypes such as β1 or θ.

ABTS substrate has been used in previous DNAzyme-LAMP studies [35,36]. In our study with cDDLAMP assay, TMB was used as DNAzyme substrate as the substrate was shown to have a higher sensitivity and faster reaction rate compared to ABTS [88]. The LOD of our cDDLAMP assay in pure culture was estimated to be 1–100 CFU/mL which is comparable to that of other STEC LAMP assays reported in previous studies [23,24,41,89–94]. This sensitivity of cDDLAMP assay is enough for practical applications such as detection of STEC in vegetable rinse water, as the infectious dose of *E. coli* O157:H7 for humans is reported to be10^1^–10^2^ CFU/mL [7,11].

cDDLAMP achieves sensitivity comparable previously reported LOD of 4.1 x10^4^ CFU/ml for *E*. *coli* O157 in raw milk using LAMP [91], and 2.26×10^0^ CFU/mL *E. coli* O157:H7 using RT-LAMP and visual LAMP [94]. However, our assay has the advantage of a 30-minute enrichment instead of a ≥3-hour culture enrichment [91]. Additionally, this method can detect low levels of STEC (10^0^ CFU/g targeting *stx1* and 10^3^ CFU/g targeting *stx2 and eae* in spiked lettuce with a LOD lower than that previously reported [23].

In comparison to commercial diagnostic kits, such as the Loopamp *Escherichia coli* O157 Detection Kit and Thermo Scientific™ SureTect™ *Escherichia coli* O157:H7 and STEC Screening PCR Assay, which offer valuable detection capabilities, there are certain limitations. These include longer enrichment times (8–24 hours) and the need for specialized equipment (Loopamp Realtime Turbidimeter or Real-Time PCR instruments), which may not be readily available in resource-limited settings. Furthermore, the Loopamp kit’s sensitivity is limited to detecting 60 CFU/test, reducing its effectiveness for detecting low bacterial concentrations. In contrast, our method utilizes a 30-minute enrichment with streptavidin magnetic beads (SMB-PAb), followed by rapid results within 30 minutes after loading the gDNA sample into a heating block, which is much faster than the 60–80 minutes required by the commercial kits. Additionally, the use of a smartphone-based microplate reader (SpotXel) makes our assay cost-effective and accessible, even in settings with limited resources. Thus, our approach offers a quicker, more accessible, and potentially more sensitive alternative for STEC detection compared to current commercial diagnostic kits.

Currently, the food production system requires testing samples to be collected in larger volumes with low bacterial concentrations to avoid expensive homogenate preparation [24]. Therefore, we inoculated the surface of intact lettuce (25 g) with 10^3-9^ CFU in our study. We were able to detect the presence of 10^5^ and 10^6^ CFU/25 g without enrichment for *stx1,* and *eae* genes, respectively, similar to previous studies [24,48]. However, IMS significantly improved the LOD by ten to one hundred-fold for *stx1* and *eae* genes, respectively (10^4^ CFU/25g). Furthermore, a strong and significant correlation of the color signal detection between smartphone-based SpotXel reader and the microplate reader with the accurate clustering of the positive and negative signal readout by each reader, indicating great promise for transforming POC diagnostics, offering a convenient, rapid, and accessible solution for the signal readout and analysis as previously reported DNAzyme-LAMP for SARS-CoV-2 detection [95]. The inconsistency between replicates in our study at extremely low cell counts could be due to negative or poor amplification efficiency of LAMP associated with low template copy numbers [96,97], resulting in increased standard deviation when compared to higher copy numbers [98,99].

Despite the ultra-sensitive detection of *stx1* and *eae* STEC genes with cDDLAMP, neither magnetic bead enrichment (10^9^ CFU/25g), nor culturing (up to 100-fold; 100 CFU/mL) resulted in the same sensitivity with *stx2* gene detection. Studies evaluating STEC detection have shown influence on the accuracy and sensitivity of the *stx* target gene due to several factors: (i) potential mismatches of LAMP primer sequences between the strain and *stx2* gene used for primer design [23,48], (ii) the presence of bacteriophage [100], (iii) DNA purity [65,101], and loss of *stx*-coding phage after sub-cultivation [100,102–104] with *stx2* loss more commonly reported [102,104,105]. Moreover, a study found *stx* instability in STEC O157: H7 isolates(n=14), and confirmed *stx* loss through whole genome sequencing and PCR results [106].

The cDDLAMP assay is a cost-effective method for detecting STEC strains without expensive machines or extraction methods, making it suitable for resource-limited locations. It incorporates two types of DNAzymes and improves sensitivity with magnetic bead enrichment. Future research should incorporate a diverse range of STEC clinical isolates with varying *eae*, and *stx2* subtypes to validate the assay’s effectiveness for broad-spectrum STEC detection in food samples. Additionally, such studies would contribute to a deeper understanding of the genetic heterogeneity and subtype variability of *eae* among non-O157 STEC strains.

## Supporting information

S1 Fig cDDLAMP assay obtained by rcDNAzyme modifications in all three primer set candidates targeting *stx1*, *stx2*, and *eae* genes. Positions of rcDNAzyme (EAD2, and DZ-00) in the sequences of FIP/BIP compared with normal primers (LAMP assay).
(TIF)

S2 Fig Photograph of the corresponding well plate reflecting the progressive improvement of the cDDLAMP reaction condition based on the optimization ofprimer concentration (A), master mix (B), and temperature (C). The photo demonstrates the corresponding changes in absorption based on reaction optimization.
(TIF)

S3 Fig Evaluation of cDDLAMP using 3different primer sets targeting stx2 gene of *E. coli* O157:H7 with varying distance between the outer primers (F3/B3): 258 bp, 229 bp, and 214 bp. All reactions were evaluated by colorimetric change with a representative photograph of well plate shown. Means from three independent experiments are shown with error bars representing standard error of the means (****P < 0.0001, and ns p > 0.05).
(TIF)

S4 Fig Photograph of the corresponding well plate reflecting the specificity of cDDLAMP of all target genes (*stx1*, *stx2*, and *eae* genes) colorimetrically and by gel electrophoresis detection of the active dual DNAzyme LAMP amplicons. The tested STEC strains are designated as *E. coli* O157:H7, O111:H8, O26:H11, and O103:H11.
(TIF)

S5 Fig Photograph of the corresponding well plate reflecting the color signal of cDDLAMP of all target genes (*stx1*, *stx2*, and *eae* genes). (A). Gel electrophoresis detection of the active dual DNAzyme LAMP amplicons (B). Gel electrophoresis of the PCR amplicons of all target genes (*stx1*, *stx2*, and *eae* genes) (C). Tested STEC strains are labeled as O157:H7, O45:H2, O145:NT, and O121:H19.
(TIF)

S6 Fig Gel electrophoresis of the PCR amplicons 247 bp, 258 bp, and 203 bp of *stx1*, *stx2*, and *eae* genes, respectively. The tested STEC strains are designated as *E. coli* O157:H7, O111:H8, O26:H11, and O103:H11.
(TIF)

S7 Fig Photograph of the corresponding well plate reflecting cDDLAMP detection of artificially inoculated lettuce by *E. coli* O157:H7 (10^3-9^ CFU/25g) without SMB-PAb enrichment (A) or with SMB-PAb enrichment (B). (TIF)

S1 Table Oligonucleotide primers used in the cDDLAMP assay. rcDNAzyme sequence (Dz-00 in BIP, and EAD2 in FIP) is underlined. (DOCX)

S2 Table Colorimetric signal to noise ratio results of SpotXel reader and microplate reader (OD655nm) with A). *E. coli* O157:H7 spiked concentration 5.2 × 10^5,3,0^ CFU/ g or mL of food matrices (#1–72) and B) *E. coli* O157:H7 spiked concentration 10^9-3^ CFU/25g with or without SMB-PAb enrichment (#1–144). (DOCX)
