# Supplementary figures and images for: Colorimetric dual DNAzyme reaction triggered by loop-mediated isothermal amplification for the visual detection of Shiga toxin-producing Escherichia coli in food matrices
Source: PLoS One. 2025 Apr 23;20(4):e0320393. doi: 10.1371/journal.pone.0320393 (PMC12017578; doi:10.1371/journal.pone.0320393)

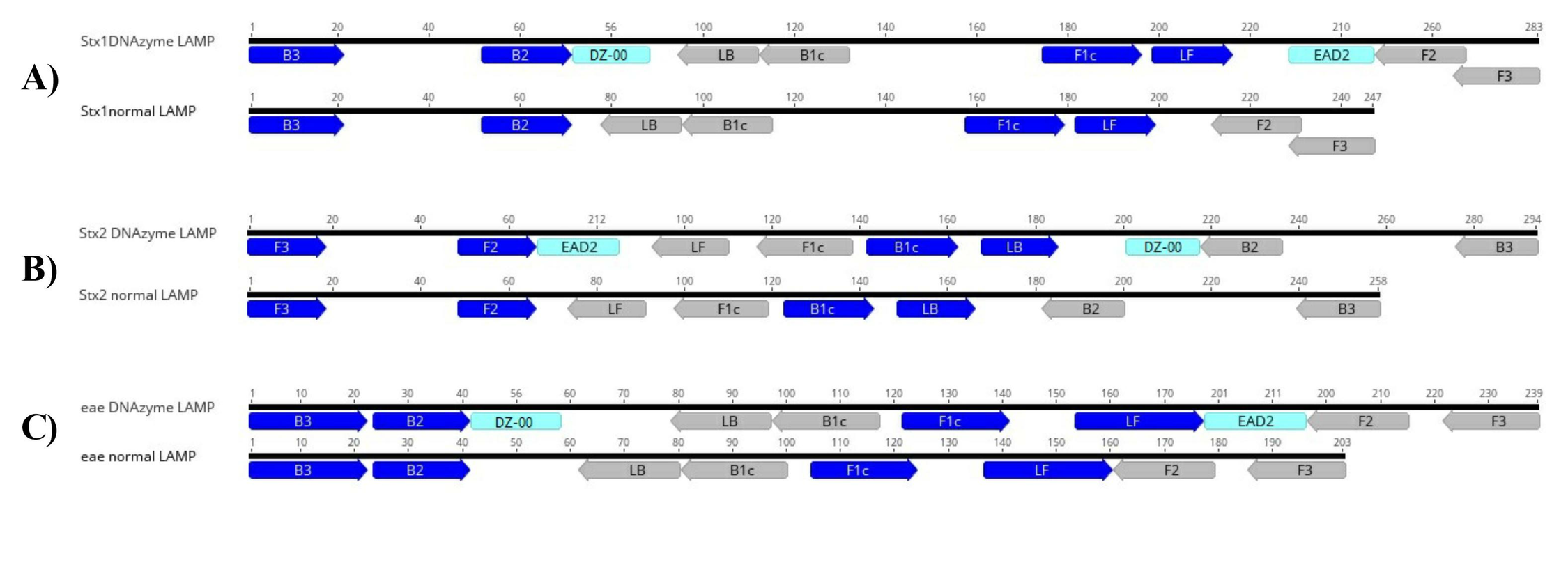

Supplement: S1 Fig — Positions of rcDNAzyme (EAD2, and DZ-00) in the sequences of FIP/BIP compared with normal primers (LAMP assay). (TIF) [file pone.0320393.s001.tif]

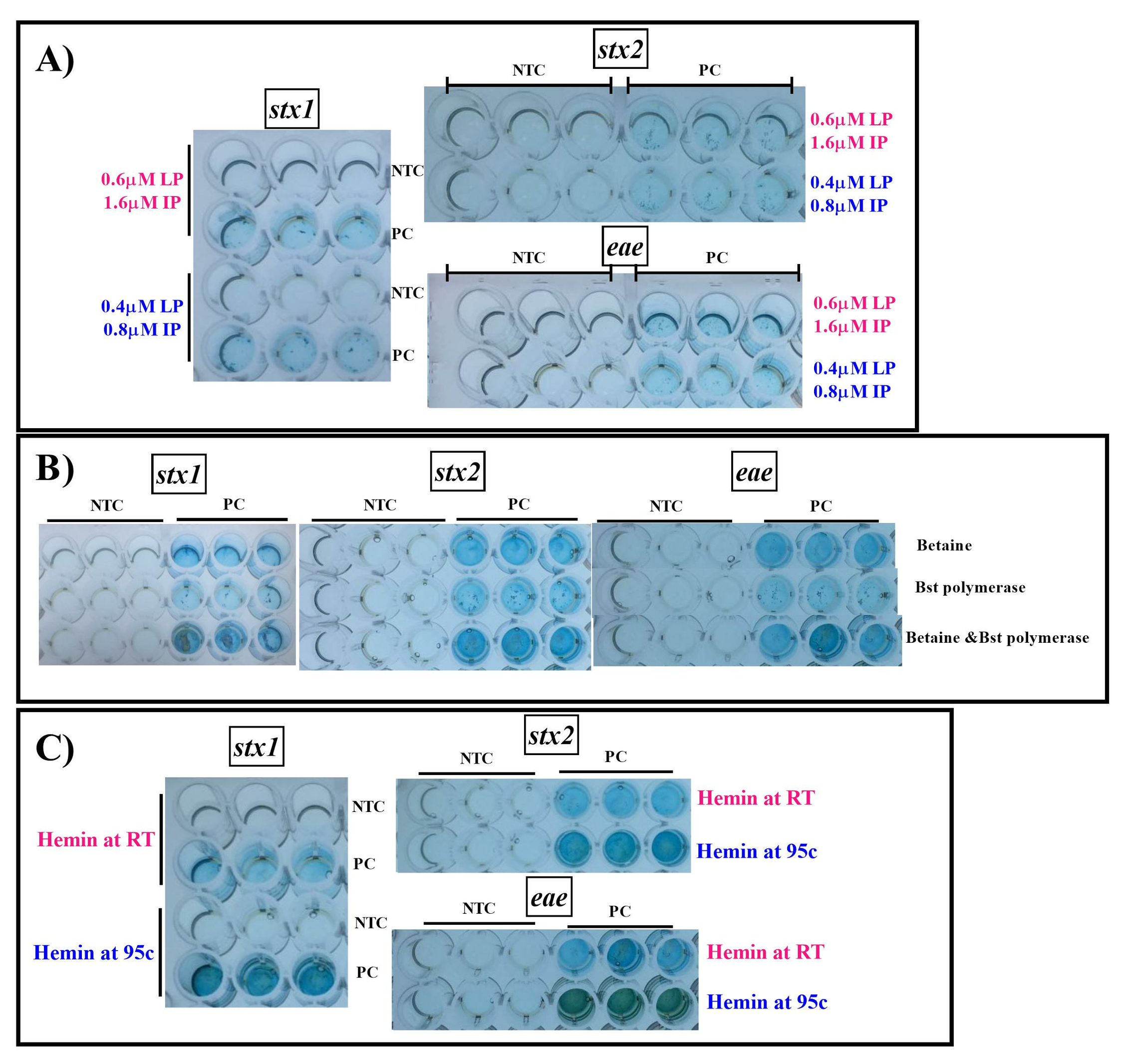

Supplement: S2 Fig — The photo demonstrates the corresponding changes in absorption based on reaction optimization. (TIF) [file pone.0320393.s002.tif]

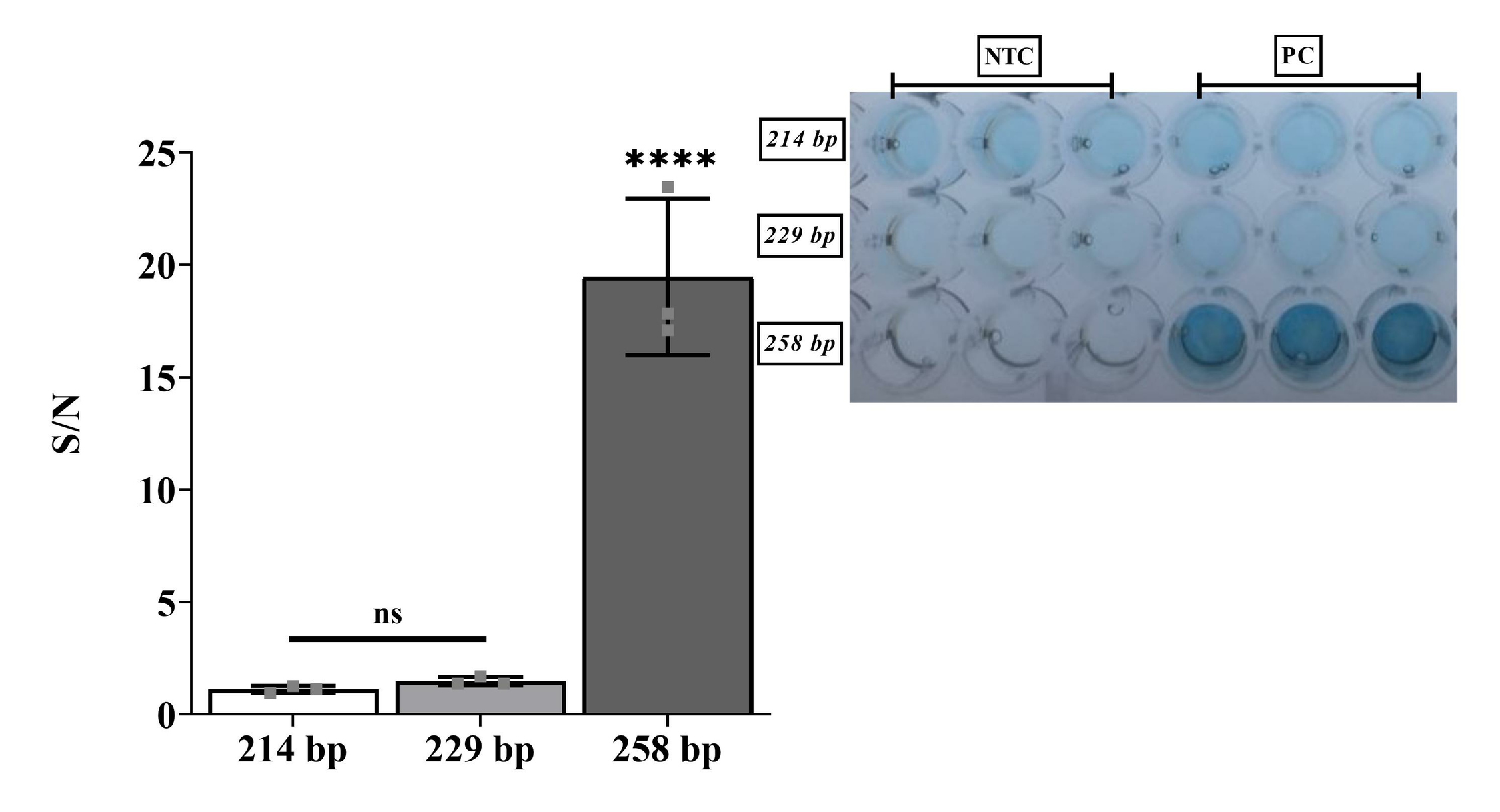

Supplement: S3 Fig — All reactions were evaluated by colorimetric change with a representative photograph of well plate shown. Means from three independent experiments are shown with error bars representing standard error of the means (****P < 0.0001, and ns p > 0.05). (TIF) [file pone.0320393.s003.tif]

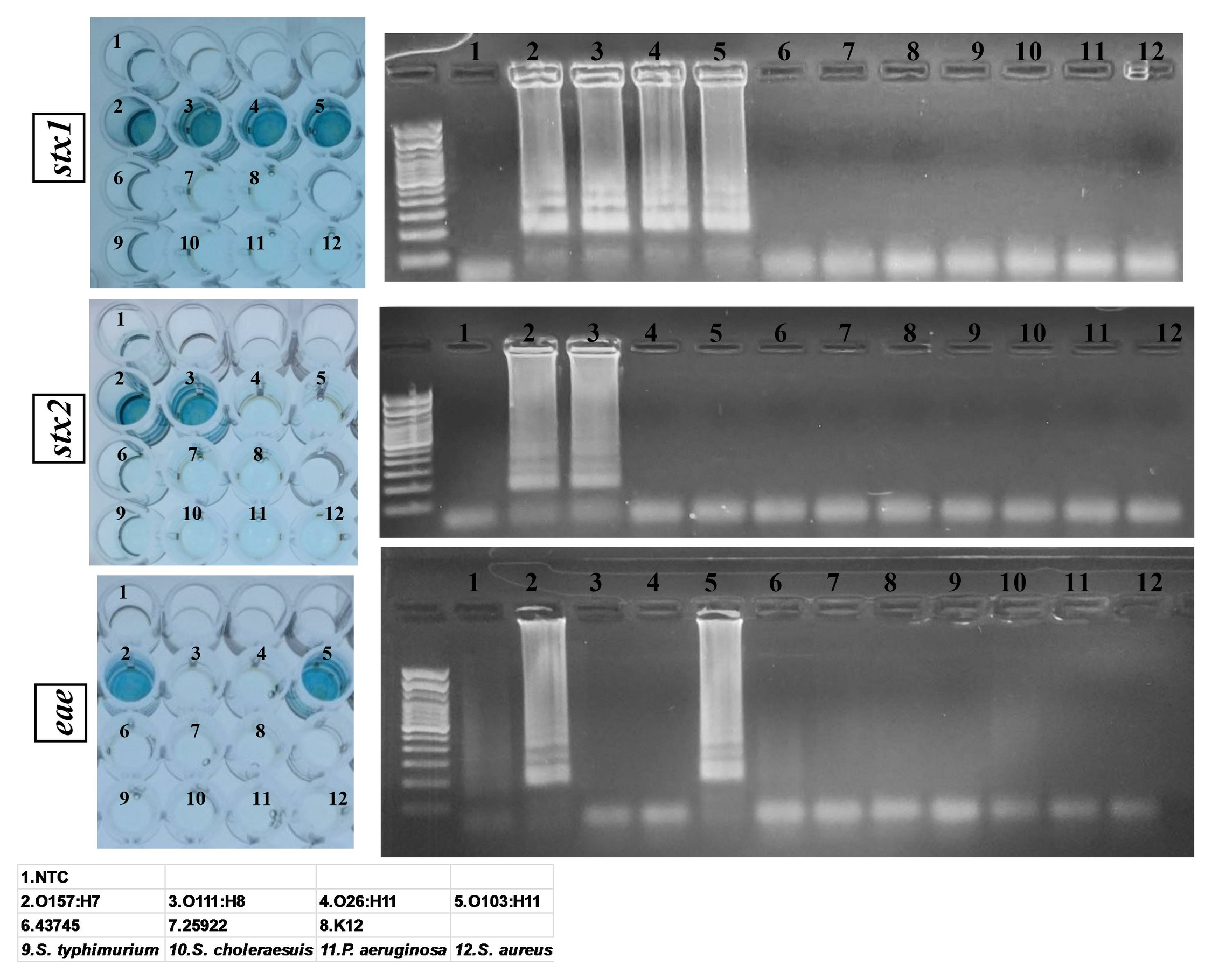

Supplement: S4 Fig — The tested STEC strains are designated as E. coli O157:H7, O111:H8, O26:H11, and O103:H11. (TIF) [file pone.0320393.s004.tif]

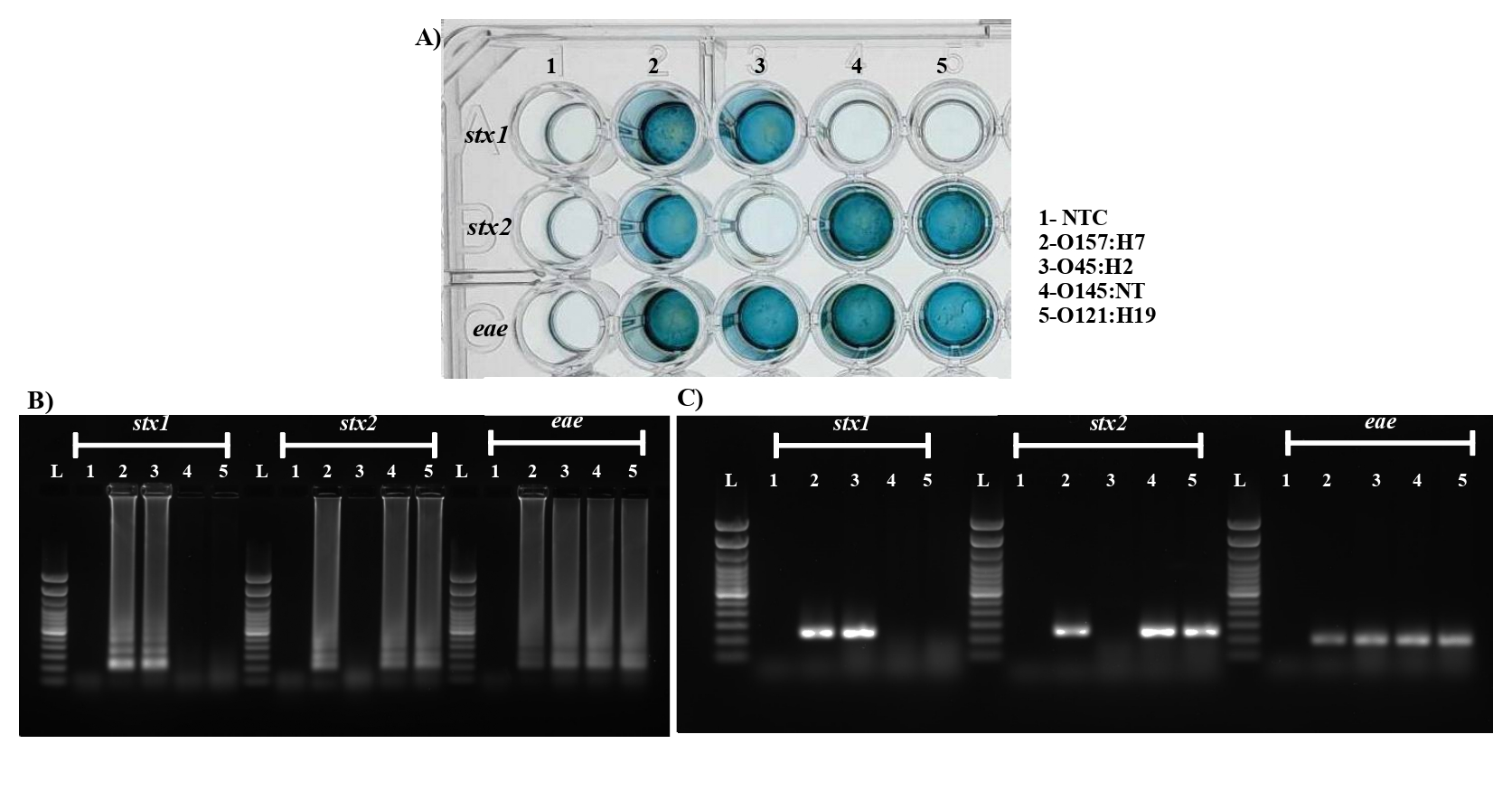

Supplement: S5 Fig — (A). Gel electrophoresis detection of the active dual DNAzyme LAMP amplicons (B). Gel electrophoresis of the PCR amplicons of all target genes (stx1, stx2, and eae genes) (C). Tested STEC strains are labeled as O157:H7, O45:H2, O145:NT, and O121:H19. (TIF) [file pone.0320393.s005.tif]

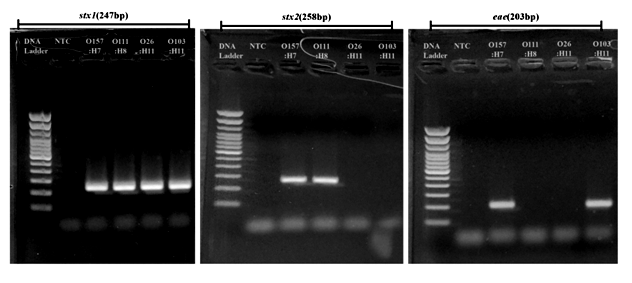

Supplement: S6 Fig — The tested STEC strains are designated as E. coli O157:H7, O111:H8, O26:H11, and O103:H11. (TIF) [file pone.0320393.s006.tif]

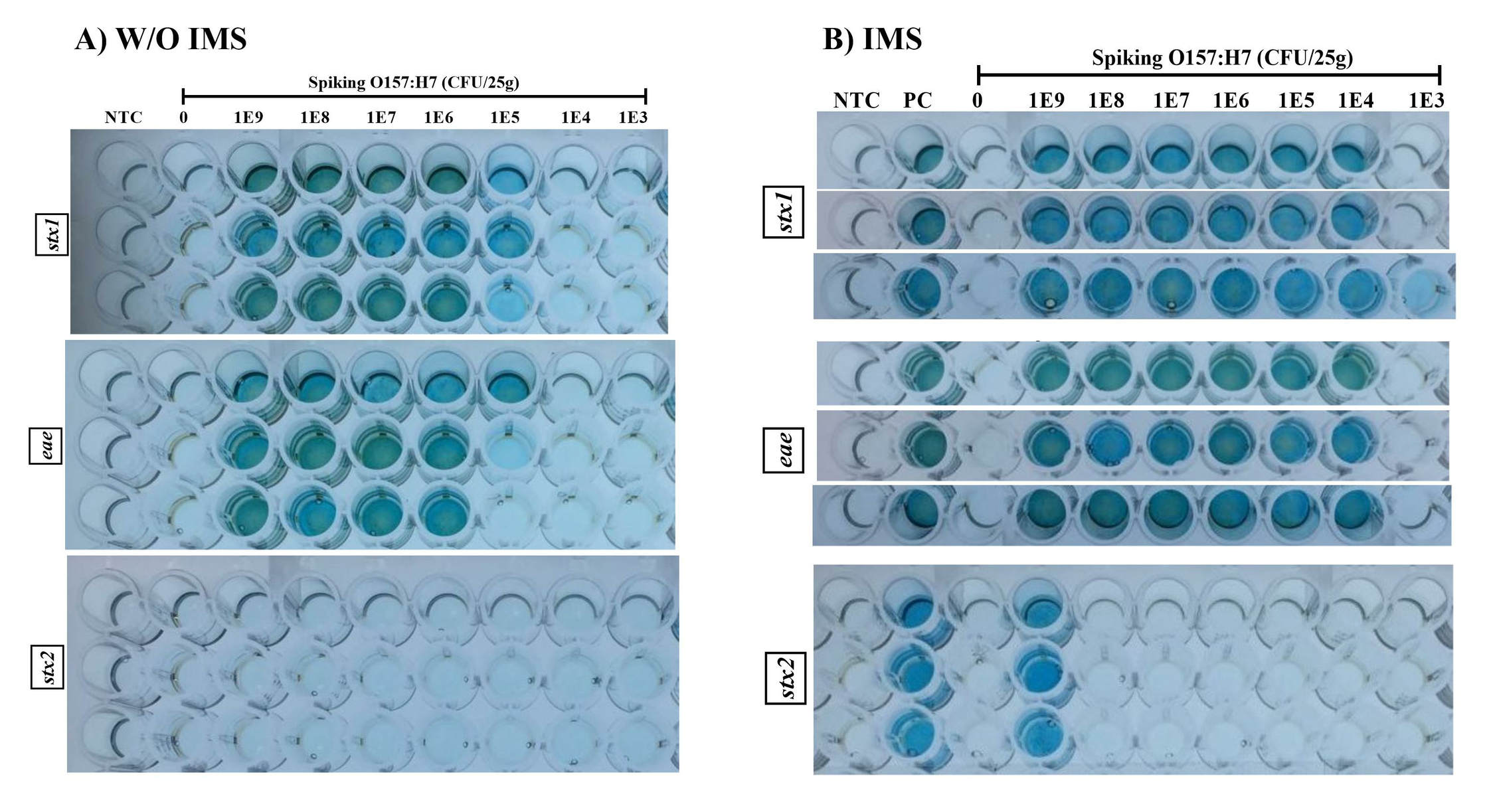

Supplement: S7 Fig — (TIF) [file pone.0320393.s007.tif]
