# Supplementary material for: Colorimetric dual DNAzyme reaction triggered by loop-mediated isothermal amplification for the visual detection of Shiga toxin-producing Escherichia coli in food matrices
Source: PLoS One. 2025 Apr 23;20(4):e0320393. doi: 10.1371/journal.pone.0320393 (PMC12017578; doi:10.1371/journal.pone.0320393)
Supplement: S1 Table — (DOCX) [file pone.0320393.s008.docx]

**S1 Table**

| Primers | Sequence | References |
| --- | --- | --- |
| *stx1*-F3 | ACAACAGCGGTTACATTGT | [40] |
| *stx1*-B3 | GATCATCCAGTGTTGTACGAA |  |
| *stx1*-FIP(EAD2) | GCGATTTATCTGCATCCCCGTACTCCCTCCCTCCCTCCCAGTGTCTGGTGACAGTAGCTAT |  |
| *stx1*-FIP(normal) | GCGATTTATCTGCATCCCCGTA-TGTCTGGTGACAGTAGCTAT |  |
| *stx1*-BIP  (Dz-00) | GGAACCTCACTGACGCAGTCCCCATCCCGCCCAACCCCTTCAGCTGTCACAGTAACA |  |
| *stx1*-BIP(normal) | GGAACCTCACTGACGCAGTC-CTTCAGCTGTCACAGTAACA |  |
| *stx1*-LF | ACTGATCCCTGCAACACG |  |
| *stx1*-LB | TGTGGCAAGAGCGATGTT |  |
| *stx2*-F3 | GCATCCAGAGCAGTTCTG |  |
| *stx2*-B3 | CAGTATAACGGCCACAGTC |  |
| *stx2*-FIP(EAD2) | GGCGTCATCGTATACACAGGAGCTCCCTCCCTCCCTCCCAGCGCTTCAGGCAGATACAG |  |
| *stx2*-FIP(normal) | GGCGTCATCGTATACACAGGAG-CGCTTCAGGCAGATACAG |  |
| *stx2*-BIP  (Dz-00) | AGACGTGGACCTCACTCTGAACCCATCCCGCCCAACCCACTCTGACACCATCCTCTC |  |
| *stx2*-BIP(normal) | AGACGTGGACCTCACTCTGAA-ACTCTGACACCATCCTCTC |  |
| *stx2*-LF | CAGACAGTGCCTGACGAA |  |
| *stx2*-LB | GGCGAATCAGCAATGTGC |  |
| *eae*-F3 | TGACTAAAATGTCCCCGG | [23] |
| *eae*-B3 | CGTTCCATAATGTTGTAACCAG |  |
| *eae*-FIP(EAD2) | GAAGCTGGCTACCGAGACTCCTCCCTCCCTCCCTCCCAGCCAAAAGCAACATGACCGA |  |
| *eae*-FIP(normal) | GAAGCTGGCTACCGAGACTC-CCAAAAGCAACATGACCGA |  |
| *eae*-BIP(Dz-00) | GCGATCTCTGAACGGCGATTCCCATCCCGCCCAACCCCCTGCAACTGTGACGAAG |  |
| *eae*-BIP(normal) | GCGATCTCTGAACGGCGATT-CCTGCAACTGTGACGAAG |  |
| *eae*-LF | GCCGCATAATTTAATGCCTTGTCA |  |
| *eae*-LB | ACGCGAAAGATACCGCTCT |  |
| *stx2*-F3 | TCGGTGTCTGTTATTAACCA | [41] |
| *stx2*-B3 | TGGAAACCGTTGTCACAC |  |
| *stx2*-FIP(EAD2) | AGACGAAGATGGTCAAAACGCCTCCCTCCCTCCCTCCCAGGCAGTTATTTTGCTGTGGA |  |
| *stx2*-BIP(Dz-00) | CCGGGTTCGTTAATACGGCACCCATCCCGCCCAACCCCGGGCACTGATATATGTGT |  |
| *stx2*-LF | TGATAGACATCAAGCCCTCGT |  |
| *stx2*-LB | CAAATACTTTCTACCGTTTT |  |
| *stx2*-F3 | CGCTTCAGGCAGATACAGAG | [23] |
| *stx2*-B3 | CCCCCTGATGATGGCAATT |  |
| *stx2*-FIP(EAD2) | TTCGCCCCCAGTTCAGAGTGACTCCCTCCCTCCCTCCCAGGTCAGGCACTGTCTGAAACT |  |
| *stx2*-BIP(Dz-00) | TGCTTCCGGAGTATCGGGGAGCCCATCCCGCCCAACCCCAGTCCCCAGTATCGCTGA |  |
| *stx2*-LF | GCGTCATCGTATACACAGGAGC |  |
| *stx2*-LB | GATGGTGTCAGAGTGGGGAGAA |  |
