# Supplementary material for: Colorimetric dual DNAzyme reaction triggered by loop-mediated isothermal amplification for the visual detection of Shiga toxin-producing Escherichia coli in food matrices
Source: PLoS One. 2025 Apr 23;20(4):e0320393. doi: 10.1371/journal.pone.0320393 (PMC12017578; doi:10.1371/journal.pone.0320393)
Supplement: S2 Table — (DOCX) [file pone.0320393.s009.docx]

**S2 Table**

| A | | | | | | |
| --- | --- | --- | --- | --- | --- | --- |
| Number | Spikedconcentration | OD | SpotXel | Sample | Gene | Food matrics |
| 1 | 0 | 1.130435 | 2.2439024 | 1 | *stx1* | milk |
| 2 | 5.20E+05 | 12.52174 | 12.323171 | 1 | *stx1* | milk |
| 3 | 5.20E+03 | 18.6087 | 9.4512195 | 1 | *stx1* | milk |
| 4 | 5.20E+00 | 1 | 3.0328947 | 1 | *stx1* | milk |
| 5 | 0 | 0.954545 | 1.2013652 | 2 | *stx1* | milk |
| 6 | 5.20E+05 | 12.90909 | 7.0511945 | 2 | *stx1* | milk |
| 7 | 5.20E+03 | 10.22727 | 7.3924915 | 2 | *stx1* | milk |
| 8 | 5.20E+00 | 3.090909 | 5.0609756 | 2 | *stx1* | milk |
| 9 | 0 | 0.9 | 2.75 | 3 | *stx1* | milk |
| 10 | 5.20E+05 | 13.7 | 15.421053 | 3 | *stx1* | milk |
| 11 | 5.20E+03 | 14.55 | 15.421053 | 3 | *stx1* | milk |
| 12 | 5.20E+00 | 13.7 | 5.78157 | 3 | *stx1* | milk |
| 13 | 0 | 0.925926 | 1.8465608 | 1 | *stx2* | milk |
| 14 | 5.20E+05 | 18.18519 | 10.407407 | 1 | *stx2* | milk |
| 15 | 5.20E+03 | 14 | 8.962963 | 1 | *stx2* | milk |
| 16 | 5.20E+00 | 0.962963 | 1.0687831 | 1 | *stx2* | milk |
| 17 | 0 | 0.952381 | 1.6013986 | 2 | *stx2* | milk |
| 18 | 5.20E+05 | 24.66667 | 14.181818 | 2 | *stx2* | milk |
| 19 | 5.20E+03 | 16.57143 | 13.825175 | 2 | *stx2* | milk |
| 20 | 5.20E+00 | 0.952381 | 1.5594406 | 2 | *stx2* | milk |
| 21 | 0 | 1 | 2.5898876 | 3 | *stx2* | milk |
| 22 | 5.20E+05 | 15.85714 | 12.106742 | 3 | *stx2* | milk |
| 23 | 5.20E+03 | 10.85714 | 11.842697 | 3 | *stx2* | milk |
| 24 | 5.20E+00 | 1.047619 | 1.2078652 | 3 | *stx2* | milk |
| 25 | 0 | 0.863636 | 2.9013158 | 1 | *eae* | milk |
| 26 | 5.20E+05 | 15.95455 | 13.901316 | 1 | *eae* | milk |
| 27 | 5.20E+03 | 17.59091 | 12.664474 | 1 | *eae* | milk |
| 28 | 5.20E+00 | 4.181818 | 5.3947368 | 1 | *eae* | milk |
| 29 | 0 | 0.666667 | 3.0065789 | 2 | *eae* | milk |
| 30 | 5.20E+05 | 18.94444 | 15.585526 | 2 | *eae* | milk |
| 31 | 5.20E+03 | 18.77778 | 14.342105 | 2 | *eae* | milk |
| 32 | 5.20E+00 | 4.444444 | 8.0505618 | 2 | *eae* | milk |
| 33 | 0 | 0.882353 | 1.0337079 | 3 | *eae* | milk |
| 34 | 5.20E+05 | 19.29412 | 10.41573 | 3 | *eae* | milk |
| 35 | 5.20E+03 | 19.35294 | 8.6292135 | 3 | *eae* | milk |
| 36 | 5.20E+00 | 16.41176 | 12.289474 | 3 | *eae* | milk |
| 37 | 0 | 0.333333 | 1.319277 | 1 | *stx1* | lettuce |
| 38 | 5.20E+05 | 10.44444 | 12.596386 | 1 | *stx1* | lettuce |
| 39 | 5.20E+03 | 8.472222 | 10.692771 | 1 | *stx1* | lettuce |
| 40 | 5.20E+00 | 8.055556 | 7.6024096 | 1 | *stx1* | lettuce |
| 41 | 0 | 0.416667 | 1.482955 | 2 | *stx1* | lettuce |
| 42 | 5.20E+05 | 13.625 | 10.227273 | 2 | *stx1* | lettuce |
| 43 | 5.20E+03 | 14.58333 | 9.6875 | 2 | *stx1* | lettuce |
| 44 | 5.20E+00 | 11.33333 | 7.0284091 | 2 | *stx1* | lettuce |
| 45 | 0 | 0.828571 | 1.471698 | 3 | *stx1* | lettuce |
| 46 | 5.20E+05 | 15.97143 | 13.283019 | 3 | *stx1* | lettuce |
| 47 | 5.20E+03 | 12.68571 | 10.830189 | 3 | *stx1* | lettuce |
| 48 | 5.20E+00 | 8.428571 | 7.4402516 | 3 | *stx1* | lettuce |
| 49 | 0 | 0.7 | 1.326203 | 1 | *stx2* | lettuce |
| 50 | 5.20E+05 | 16.6 | 11.208556 | 1 | *stx2* | lettuce |
| 51 | 5.20E+03 | 12.15 | 4.7112299 | 1 | *stx2* | lettuce |
| 52 | 5.20E+00 | 0.8 | 1.6951872 | 1 | *stx2* | lettuce |
| 53 | 0 | 0.769231 | 1.298343 | 2 | *stx2* | lettuce |
| 54 | 5.20E+05 | 21.53846 | 11.596685 | 2 | *stx2* | lettuce |
| 55 | 5.20E+03 | 12.61538 | 6.0497238 | 2 | *stx2* | lettuce |
| 56 | 5.20E+00 | 0.846154 | 1.5082873 | 2 | *stx2* | lettuce |
| 57 | 0 | 0.848485 | 1.493421 | 3 | *stx2* | lettuce |
| 58 | 5.20E+05 | 12.12121 | 10.092105 | 3 | *stx2* | lettuce |
| 59 | 5.20E+03 | 6.909091 | 6.7434211 | 3 | *stx2* | lettuce |
| 60 | 5.20E+00 | 0.818182 | 1.6315789 | 3 | *stx2* | lettuce |
| 61 | 0 | 0.888889 | 1.043011 | 1 | *eae* | lettuce |
| 62 | 5.20E+05 | 18.83333 | 9.5537634 | 1 | *eae* | lettuce |
| 63 | 5.20E+03 | 18.16667 | 8.6182796 | 1 | *eae* | lettuce |
| 64 | 5.20E+00 | 10.61111 | 7.0806452 | 1 | *eae* | lettuce |
| 65 | 0 | 0.666667 | 1.124324 | 2 | *eae* | lettuce |
| 66 | 5.20E+05 | 20.06667 | 9.3783784 | 2 | *eae* | lettuce |
| 67 | 5.20E+03 | 20.13333 | 7.3405405 | 2 | *eae* | lettuce |
| 68 | 5.20E+00 | 0.733333 | 1.8864865 | 2 | *eae* | lettuce |
| 69 | 0 | 0.642857 | 0.979167 | 3 | *eae* | lettuce |
| 70 | 5.20E+05 | 14.5 | 9.2447917 | 3 | *eae* | lettuce |
| 71 | 5.20E+03 | 19.07143 | 8.34375 | 3 | *eae* | lettuce |
| 72 | 5.20E+00 | 0.714286 | 1.109375 | 3 | *eae* | lettuce |

| B | | | | | | |
| --- | --- | --- | --- | --- | --- | --- |
| Number | Spikedconcentration | OD | SpotXel | sample | Gene | Enrichment |
| 1 | 0 | 1.5 | 1.4766355 | 1 | *stx1* | IMS |
| 2 | 1.00E+09 | 20.0625 | 9.5514019 | 1 | *stx1* | IMS |
| 3 | 1.00E+08 | 19.4375 | 9.8130841 | 1 | *stx1* | IMS |
| 4 | 1.00E+07 | 19.3125 | 8.7616822 | 1 | *stx1* | IMS |
| 5 | 1.00E+06 | 17.625 | 8.4719626 | 1 | *stx1* | IMS |
| 6 | 1.00E+05 | 17.4375 | 7.8878505 | 1 | *stx1* | IMS |
| 7 | 1.00E+04 | 16.8125 | 7.9205607 | 1 | *stx1* | IMS |
| 8 | 1.00E+03 | 1.5625 | 3.9953271 | 1 | *stx1* | IMS |
| 9 | 0 | 0.678571 | 1.4339623 | 2 | *stx1* | IMS |
| 10 | 1.00E+09 | 13.25 | 8.2301887 | 2 | *stx1* | IMS |
| 11 | 1.00E+08 | 12 | 7.0264151 | 2 | *stx1* | IMS |
| 12 | 1.00E+07 | 11.67857 | 7.1773585 | 2 | *stx1* | IMS |
| 13 | 1.00E+06 | 10.53571 | 7.3584906 | 2 | *stx1* | IMS |
| 14 | 1.00E+05 | 10.5 | 7.0679245 | 2 | *stx1* | IMS |
| 15 | 1.00E+04 | 9.928571 | 5.7358491 | 2 | *stx1* | IMS |
| 16 | 1.00E+03 | 3.892857 | 1.6641509 | 2 | *stx1* | IMS |
| 17 | 0 | 0.875 | 1.6106195 | 3 | *stx1* | IMS |
| 18 | 1.00E+09 | 29.25 | 8.4557522 | 3 | *stx1* | IMS |
| 19 | 1.00E+08 | 29 | 8.9690265 | 3 | *stx1* | IMS |
| 20 | 1.00E+07 | 25.625 | 8.5132743 | 3 | *stx1* | IMS |
| 21 | 1.00E+06 | 25.5 | 7.3362832 | 3 | *stx1* | IMS |
| 22 | 1.00E+05 | 25 | 8.2433628 | 3 | *stx1* | IMS |
| 23 | 1.00E+04 | 23.875 | 6.6902655 | 3 | *stx1* | IMS |
| 24 | 1.00E+03 | 1.0625 | 2.4469027 | 3 | *stx1* | IMS |
| 25 | 0 | 1.785714 | 1.3859649 | 1 | *stx2* | IMS |
| 26 | 1.00E+09 | 25.46429 | 12.730994 | 1 | *stx2* | IMS |
| 27 | 1.00E+08 | 1.357143 | 1.4561404 | 1 | *stx2* | IMS |
| 28 | 1.00E+07 | 1.285714 | 1.1111111 | 1 | *stx2* | IMS |
| 29 | 1.00E+06 | 1.678571 | 0.8479532 | 1 | *stx2* | IMS |
| 30 | 1.00E+05 | 1.571429 | 0.6432749 | 1 | *stx2* | IMS |
| 31 | 1.00E+04 | 1.285714 | 0.7836257 | 1 | *stx2* | IMS |
| 32 | 1.00E+03 | 1.214286 | 0.8421053 | 1 | *stx2* | IMS |
| 33 | 0 | 0.961538 | 1.0625 | 2 | *stx2* | IMS |
| 34 | 1.00E+09 | 30.42308 | 13.401042 | 2 | *stx2* | IMS |
| 35 | 1.00E+08 | 1.615385 | 1.3489583 | 2 | *stx2* | IMS |
| 36 | 1.00E+07 | 1.538462 | 0.8072917 | 2 | *stx2* | IMS |
| 37 | 1.00E+06 | 1.692308 | 0.7395833 | 2 | *stx2* | IMS |
| 38 | 1.00E+05 | 1.692308 | 0.8229167 | 2 | *stx2* | IMS |
| 39 | 1.00E+04 | 1.538462 | 0.8958333 | 2 | *stx2* | IMS |
| 40 | 1.00E+03 | 1.576923 | 0.5625 | 2 | *stx2* | IMS |
| 41 | 0 | 1.238095 | 2.6057692 | 3 | *stx2* | IMS |
| 42 | 1.00E+09 | 28.09524 | 18.375 | 3 | *stx2* | IMS |
| 43 | 1.00E+08 | 1.714286 | 2.9134615 | 3 | *stx2* | IMS |
| 44 | 1.00E+07 | 1.52381 | 1.2596154 | 3 | *stx2* | IMS |
| 45 | 1.00E+06 | 1.571429 | 1.5192308 | 3 | *stx2* | IMS |
| 46 | 1.00E+05 | 1.52381 | 1.7211538 | 3 | *stx2* | IMS |
| 47 | 1.00E+04 | 1.571429 | 1.1442308 | 3 | *stx2* | IMS |
| 48 | 1.00E+03 | 1.619048 | 1.7788462 | 3 | *stx2* | IMS |
| 49 | 0 | 1 | 2.2261905 | 1 | *eae* | IMS |
| 50 | 1.00E+09 | 18.95238 | 11.505952 | 1 | *eae* | IMS |
| 51 | 1.00E+08 | 17.57143 | 11.113095 | 1 | *eae* | IMS |
| 52 | 1.00E+07 | 17.42857 | 7.0714286 | 1 | *eae* | IMS |
| 53 | 1.00E+06 | 16.42857 | 9.2142857 | 1 | *eae* | IMS |
| 54 | 1.00E+05 | 15.42857 | 8.6964286 | 1 | *eae* | IMS |
| 55 | 1.00E+04 | 15.09524 | 5.3035714 | 1 | *eae* | IMS |
| 56 | 1.00E+03 | 1.047619 | 3.5535714 | 1 | *eae* | IMS |
| 57 | 0 | 1.055556 | 2.0630631 | 2 | *eae* | IMS |
| 58 | 1.00E+09 | 26.61111 | 21.441441 | 2 | *eae* | IMS |
| 59 | 1.00E+08 | 25.5 | 16.837838 | 2 | *eae* | IMS |
| 60 | 1.00E+07 | 25.05556 | 14.702703 | 2 | *eae* | IMS |
| 61 | 1.00E+06 | 21.38889 | 13.396396 | 2 | *eae* | IMS |
| 62 | 1.00E+05 | 20.66667 | 9.2522523 | 2 | *eae* | IMS |
| 63 | 1.00E+04 | 18.05556 | 7.990991 | 2 | *eae* | IMS |
| 64 | 1.00E+03 | 1 | 4.5675676 | 2 | *eae* | IMS |
| 65 | 0 | 1.066667 | 1.3838384 | 3 | *eae* | IMS |
| 66 | 1.00E+09 | 33.4 | 10.060606 | 3 | *eae* | IMS |
| 67 | 1.00E+08 | 31.8 | 9.6717172 | 3 | *eae* | IMS |
| 68 | 1.00E+07 | 31.13333 | 7.5909091 | 3 | *eae* | IMS |
| 69 | 1.00E+06 | 30.73333 | 7.6616162 | 3 | *eae* | IMS |
| 70 | 1.00E+05 | 29.86667 | 6.8484848 | 3 | *eae* | IMS |
| 71 | 1.00E+04 | 28.73333 | 4.6717172 | 3 | *eae* | IMS |
| 72 | 1.00E+03 | 0.933333 | 2.8939394 | 3 | *eae* | IMS |
| 73 | 0 | 1.235294 | 1.354717 | 1 | *stx1* | No IMS |
| 74 | 1.00E+09 | 19.5 | 6.9886792 | 1 | *stx1* | No IMS |
| 75 | 1.00E+08 | 18.61765 | 7.0226415 | 1 | *stx1* | No IMS |
| 76 | 1.00E+07 | 15.94118 | 4.7660377 | 1 | *stx1* | No IMS |
| 77 | 1.00E+06 | 13.52941 | 4.4339623 | 1 | *stx1* | No IMS |
| 78 | 1.00E+05 | 9.176471 | 4.490566 | 1 | *stx1* | No IMS |
| 79 | 1.00E+04 | 1.882353 | 1.9245283 | 1 | *stx1* | No IMS |
| 80 | 1.00E+03 | 1.529412 | 1.909434 | 1 | *stx1* | No IMS |
| 81 | 0 | 1.085714 | 1.2465753 | 2 | *stx1* | No IMS |
| 82 | 1.00E+09 | 19.65714 | 5.890411 | 2 | *stx1* | No IMS |
| 83 | 1.00E+08 | 17.34286 | 6.0650685 | 2 | *stx1* | No IMS |
| 84 | 1.00E+07 | 13.14286 | 4.4109589 | 2 | *stx1* | No IMS |
| 85 | 1.00E+06 | 12.11429 | 3.989726 | 2 | *stx1* | No IMS |
| 86 | 1.00E+05 | 10.4 | 5.5532995 | 2 | *stx1* | No IMS |
| 87 | 1.00E+04 | 1.942857 | 1.6643836 | 2 | *stx1* | No IMS |
| 88 | 1.00E+03 | 1.4 | 1.260274 | 2 | *stx1* | No IMS |
| 89 | 0 | 1.272727 | 1.928934 | 3 | *stx1* | No IMS |
| 90 | 1.00E+09 | 18.63636 | 9.0253807 | 3 | *stx1* | No IMS |
| 91 | 1.00E+08 | 18.93939 | 8.6954315 | 3 | *stx1* | No IMS |
| 92 | 1.00E+07 | 17.39394 | 5.8730964 | 3 | *stx1* | No IMS |
| 93 | 1.00E+06 | 14.60606 | 5.5025381 | 3 | *stx1* | No IMS |
| 94 | 1.00E+05 | 5.363636 | 3.7328767 | 3 | *stx1* | No IMS |
| 95 | 1.00E+04 | 1.636364 | 2.6192893 | 3 | *stx1* | No IMS |
| 96 | 1.00E+03 | 1.606061 | 2.116751 | 3 | *stx1* | No IMS |
| 97 | 0 | 1.272727 | 0.7708333 | 1 | *stx2* | No IMS |
| 98 | 1.00E+09 | 2.045455 | 0.765625 | 1 | *stx2* | No IMS |
| 99 | 1.00E+08 | 1.954545 | 0.7916667 | 1 | *stx2* | No IMS |
| 100 | 1.00E+07 | 1.464286 | 0.84375 | 1 | *stx2* | No IMS |
| 101 | 1.00E+06 | 1.772727 | 0.765625 | 1 | *stx2* | No IMS |
| 102 | 1.00E+05 | 1.727273 | 0.5520833 | 1 | *stx2* | No IMS |
| 103 | 1.00E+04 | 1.818182 | 0.9895833 | 1 | *stx2* | No IMS |
| 104 | 1.00E+03 | 1.772727 | 0.963542 | 1 | *stx2* | No IMS |
| 105 | 0 | 0.709677 | 1.1575758 | 2 | *stx2* | No IMS |
| 106 | 1.00E+09 | 1.225806 | 1.2242424 | 2 | *stx2* | No IMS |
| 107 | 1.00E+08 | 1.387097 | 1.2060606 | 2 | *stx2* | No IMS |
| 108 | 1.00E+07 | 1 | 1.1515152 | 2 | *stx2* | No IMS |
| 109 | 1.00E+06 | 1 | 0.7939394 | 2 | *stx2* | No IMS |
| 110 | 1.00E+05 | 1.064516 | 0.5212121 | 2 | *stx2* | No IMS |
| 111 | 1.00E+04 | 1.032258 | 1.0121212 | 2 | *stx2* | No IMS |
| 112 | 1.00E+03 | 1.16129 | 0.793939 | 2 | *stx2* | No IMS |
| 113 | 0 | 1 | 0.6568047 | 3 | *stx2* | No IMS |
| 114 | 1.00E+09 | 1.269231 | 1 | 3 | *stx2* | No IMS |
| 115 | 1.00E+08 | 1.153846 | 0.9408284 | 3 | *stx2* | No IMS |
| 116 | 1.00E+07 | 1.115385 | 0.9940828 | 3 | *stx2* | No IMS |
| 117 | 1.00E+06 | 1.230769 | 1.1005917 | 3 | *stx2* | No IMS |
| 118 | 1.00E+05 | 0.884615 | 3.887574 | 3 | *stx2* | No IMS |
| 119 | 1.00E+04 | 1.307692 | 0.7869822 | 3 | *stx2* | No IMS |
| 120 | 1.00E+03 | 1.269231 | 0.940828 | 3 | *stx2* | No IMS |
| 121 | 0 | 1 | 1.1736842 | 1 | *eae* | No IMS |
| 122 | 1.00E+09 | 20.75758 | 9.0736842 | 1 | *eae* | No IMS |
| 123 | 1.00E+08 | 20.0303 | 7.5684211 | 1 | *eae* | No IMS |
| 124 | 1.00E+07 | 18.84848 | 6.6842105 | 1 | *eae* | No IMS |
| 125 | 1.00E+06 | 15.78788 | 6.1368421 | 1 | *eae* | No IMS |
| 126 | 1.00E+05 | 13.75758 | 5.9631579 | 1 | *eae* | No IMS |
| 127 | 1.00E+04 | 1.424242 | 1.9631579 | 1 | *eae* | No IMS |
| 128 | 1.00E+03 | 1.121212 | 2.336842 | 1 | *eae* | No IMS |
| 129 | 0 | 0.827586 | 1.2035928 | 2 | *eae* | No IMS |
| 130 | 1.00E+09 | 23.72414 | 9.9820359 | 2 | *eae* | No IMS |
| 131 | 1.00E+08 | 23.06897 | 8.1676647 | 2 | *eae* | No IMS |
| 132 | 1.00E+07 | 20.31034 | 8.1377246 | 2 | *eae* | No IMS |
| 133 | 1.00E+06 | 15.86207 | 7.1556886 | 2 | *eae* | No IMS |
| 134 | 1.00E+05 | 5.344828 | 5.1257485 | 2 | *eae* | No IMS |
| 135 | 1.00E+04 | 1.517241 | 2.5988024 | 2 | *eae* | No IMS |
| 136 | 1.00E+03 | 1.482759 | 2.293413 | 2 | *eae* | No IMS |
| 137 | 0 | 1.185185 | 1.2918919 | 3 | *eae* | No IMS |
| 138 | 1.00E+09 | 25.22222 | 8.0918919 | 3 | *eae* | No IMS |
| 139 | 1.00E+08 | 22.03704 | 7.5297297 | 3 | *eae* | No IMS |
| 140 | 1.00E+07 | 22.92593 | 6.3081081 | 3 | *eae* | No IMS |
| 141 | 1.00E+06 | 20.51852 | 5.5891892 | 3 | *eae* | No IMS |
| 142 | 1.00E+05 | 1.407407 | 2.7081081 | 3 | *eae* | No IMS |
| 143 | 1.00E+04 | 1.518519 | 2.1297297 | 3 | *eae* | No IMS |
| 144 | 1.00E+03 | 1.444444 | 2.091892 | 3 | *eae* | No IMS |
